# A central role for TOR signalling in a yeast model for juvenile CLN3 disease

**DOI:** 10.15698/mic2015.12.241

**Published:** 2015-11-11

**Authors:** Michael E. Bond, Rachel Brown, Charalampos Rallis, Jürg Bähler, Sara E. Mole

**Affiliations:** 1 MRC Laboratory for Molecular Cell Biology, University College London, London WC1E 6BT, UK.; 2 UCL Institute of Child Health, 30 Guilford Street, London WC1N 1EH, UK.; 3 Department of Genetics, Evolution and Environment, University College London, London WC1E 6BT, UK.; 4 Institute of Healthy Ageing, University College London, London WC1E 6BT, UK.

**Keywords:** Batten disease, NCL, CLN3, btn1, Tor, TORC, S. pombe, yeast

## Abstract

Yeasts provide an excellent genetically tractable eukaryotic system for investigating the function of genes in their biological context, and are especially relevant for those conserved genes that cause disease. We study the role of *btn1*, the orthologue of a human gene that underlies an early onset neurodegenerative disease (juvenile CLN3 disease, neuronal ceroid lipofuscinosis (NCLs) or Batten disease) in the fission yeast *Schizosaccharomyces pombe*. A global screen for genetic interactions with *btn1* highlighted a conserved key signalling hub in which multiple components functionally relate to this conserved disease gene. This signalling hub includes two major mitogen-activated protein kinase (MAPK) cascades, and centers on the Tor kinase complexes TORC1 and TORC2. We confirmed that yeast cells modelling CLN3 disease exhibit features consistent with dysfunction in the TORC pathways, and showed that modulating TORC function leads to a comprehensive rescue of defects in this yeast disease model. The same pathways may be novel targets in the development of therapies for the NCLs and related diseases.

## INTRODUCTION

Yeasts have long been used as model systems to shed light on basic eukaryotic cell biology, and can provide a rapid and comprehensive route of investigation for genes of unknown function. This is particularly relevant for those genes that are conserved across diverse eukaryotic species and which can be presumed to play a fundamental biological role. A significant health challenge facing current research and drug development is the increasing incidence of age-related neurodegenerative disorders. Elucidating the mechanisms that underlie neurodegeneration is complex, particularly so in common age-related dementias that may have numerous contributing factors. There are, however, monogenic inherited neurodegenerative diseases that present a simpler alternative for investigation and real opportunities to determine these basic cellular changes, and some of the underlying genes are conserved even in yeasts. The genetic tractability of yeasts offers particular advantages to the challenges of understanding disease mechanisms in a relevant biological context and this knowledge can inform therapeutic development for such conserved genetic diseases.

The neuronal ceroid lipofuscinoses (NCLs) are such a group of monogenic neurodegenerative disorders that generally affect children 1. The most common of these is juvenile CLN3 disease 2. This disease is characterised by progressive neuronal atrophy that causes visual failure, seizures and a progressive decline in cognitive and motor function. This disease is accompanied by cellular features characteristic of many neurodegenerative conditions 3,4 that include the dysfunction of core cellular processes, such as reduced lysosomal and autophagic clearance 5,6 and mitochondrial abnormalities 7. Moreover, CLN3 disease also leads to specific cellular pathologies characteristic of more common dementias. These include the accumulation of lipofuscin 1, as observed in aged neurons 4, aberrant amyloid-β processing 5, a feature of Alzheimer’s disease 8 and α-synuclein accumulation 9, a feature of Parkinson’s disease 10.

Juvenile CLN3 disease is caused by mutations in a single gene (*CLN3*) 2, whose function is unknown but which is highly conserved across eukaryotic species 11. As a consequence, this disease is relatively straightforward to reproduce in experimental systems and, given its similarity to more common dementias, such experimental models are ideal paradigms to study the basic cellular changes that occur in neurodegenerative diseases. The fission yeast *Schizosaccharomyces pombe* contains a single orthologue of *CLN3* (*btn1*) 11. Work in this yeast has revealed a role for *btn1* in many cellular processes. Like patient cells, yeast lacking *btn1* (*btn1*Δ) have enlarged and less acidic vacuoles 12 and further work has supported roles for *btn1* in vacuolar homeostasis 13. The ability of *btn1* modeling the most common CLN3 mutation (a 1 Kb deletion) to rescue these vacuolar defects found in the *btn1*Δ null strain has also revealed that this mutant allele retains some function, making juvenile CLN3 disease a mutation-specific disease rather than the consequence of a complete loss of function as originally assumed 14.

A host of further morphological defects exhibited in the *btn1*Δ strain support additional roles for *btn1* in cytokinesis 12 and the organisation of cell polarity 15. Furthermore, *btn1*Δ cells also display cell wall defects 16. Importantly, work in this model has expanded our understanding of the disease by revealing that *btn1* is involved in two independent pathways; one pH-dependent and one pH-independent, thus providing the first suggestion that Batten disease is more than a pH-related lysosome disorder 16. Indeed, a comprehensive metabolomics approach has revealed that *btn1* is required for the regulation of glycolysis and amino acid homeostasis 17. The involvement of *btn1* in numerous, apparently disparate, pathways may be a result of alterations at the Golgi apparatus, as the number, morphology, and location of thus organelle are affected by its deletion 13. Lastly, this model has been used successfully to model disease mutations in Btn1p, an effort that has provided valuable insight into their consequences on protein trafficking and function. It was found that equivalent CLN3 disease mutations in *btn1* affect the yeast phenotype in a way that can accurately predict the severity of disease, further establishing yeast as an accurate disease model despite its simplicity. Importantly, the observations reported in the fission yeast model have consistently been confirmed in mammalian systems 5,18,19. Unfortunately however, despite these insights, the molecular processes that underlie cell death in this disease are poorly understood, the function of *CLN3* is unknown, and there remains a significant need for protective therapeutic targets.

A particular advantage of yeast model systems is the availability of genome-wide techniques. Synthetic genetic arrays (SGAs) have proved a particularly powerful means of exploring genetic interactions in yeast species 20. This approach highlights genes involved in pathways parallel to, or converging with, the query gene. This provides information about functional relationships among genes, as well as processes that suppress the defects associated with a particular mutation. As they are hypothesis-free, SGAs are particularly valuable in the investigation of complex biological problems and those where gene function is unclear. These advantages are particularly relevant for neurodegeneration in general, due to the complexity of the problem, and for juvenile CLN3 disease in particular, due to the lack of a clear gene function. SGAs have previously been employed in budding yeast to investigate mutant huntingtin and α-synuclein toxicity 21. Such an approach can place the gene under investigation within its biological context and thereby uncover much-needed protective pathways for neurodegenerative disease.

We applied SGA analysis to identify pathways that are altered as a consequence of loss of function of *btn1* in an effort to better understand the molecular consequences of CLN3 disease, and to provide new candidate target pathways and processes for therapeutic development. A third of the genetic interactions that were identified centered on a set of conserved and connected signalling pathways. Manipulation of these pathways leads to a complete rescue of the pleiotropic array of *btn1*Δ phenotypes. This approach represents the most successful rescue of cellular dysfunction in any model for juvenile CLN3 disease to date.

## RESULTS

### Genome-wide analysis of genetic interactions with *btn1* reveals a central role for TOR kinases

**Figure 1:**
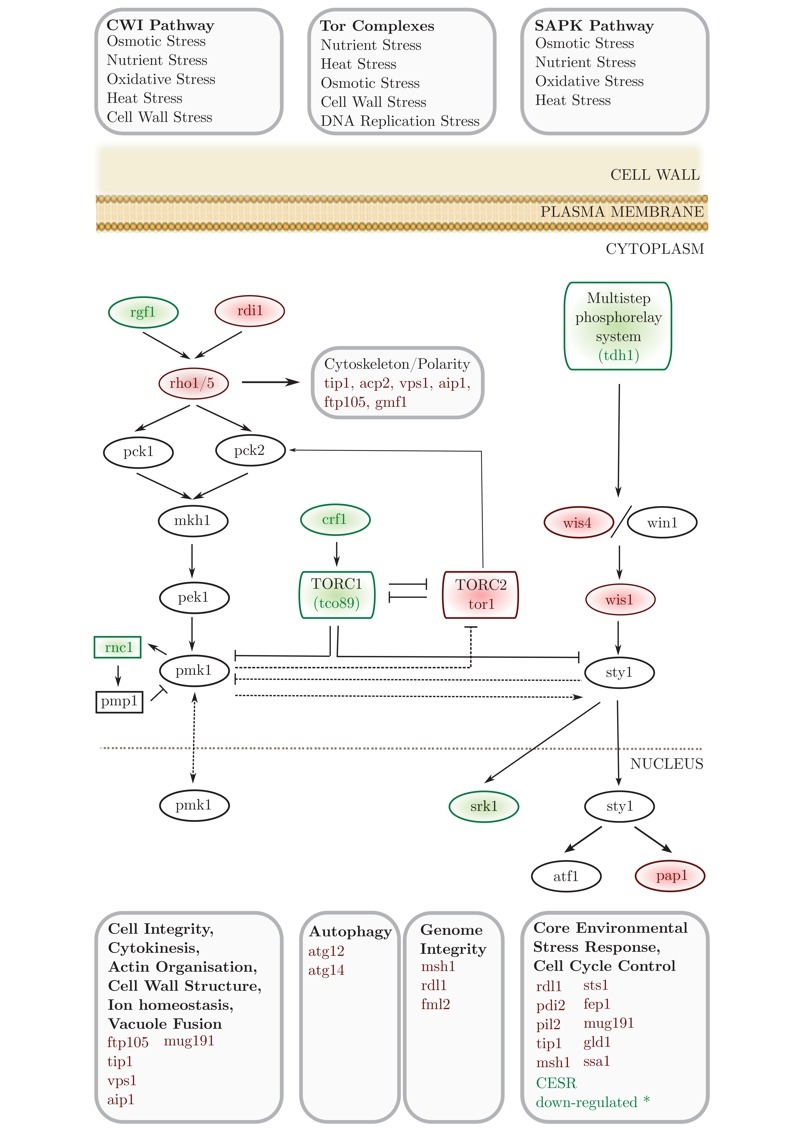
FIGURE 1: Synthetic genetic array (SGA) analysis of the genetic interactions of *btn1*. A schematic of the genes involved in stress responses that genetically interact with *btn1.* Genes in red were identified as negative interactors and those in green as positive. Grey genes are involved in these pathways but were not found to interact with *btn1.* * CESR refers to ‘core environmental stress response’ genes, a group of genes that respond to most environmental stressors as described by Chen *et al.* (2003) 76.

We applied an SGA approach as an unbiased, genome-wide strategy to probe the interactions of a conserved neurodegenerative disease gene (*btn1*). We identified 331 positive interactors and 131 negative interactors of *btn1 *(Table S1A and B). A large number of negative interactions (n = 39; 29.7%) mapped to a set of highly interconnected signalling processes, which center on the Tor kinase complexes TORC1 and TORC2 (Fig. 1). Core components of both complexes display genetic interactions with *btn1*; *tco89, *encoding a component of TORC1, was found to interact positively with *btn1,* while *tor1, *encoding the Tor kinase of TORC2, was found to interact negatively with *btn1*. Such a pattern suggests that TORC2 signalling is beneficial to the fitness of *btn1*Δ cells, whereas TORC1 signalling is detrimental, an observation that is in keeping with the idea that the two complexes play opposing roles and undergo mutual repression 22,23,24. *btn1 *was also found to negatively interact with genes encoding components of the connected cell wall integrity (CWI) and stress-associated protein kinase (SAPK) MAP kinase cascades, and a number of down-stream stress regulated genes (Table S1A and B).

### *btn1*Δ cells display features consistent with dysfunctional Tor signalling

**Figure 2:**
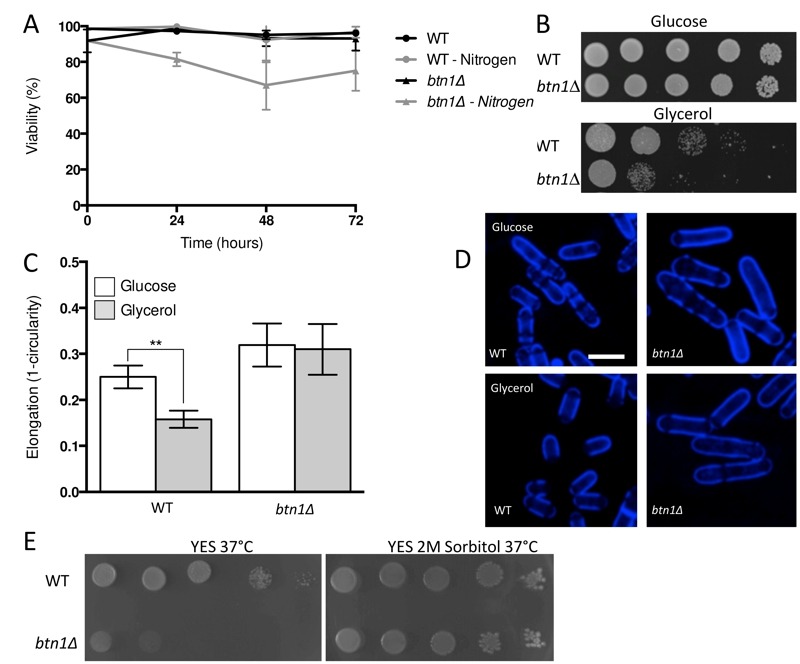
FIGURE 2: *btn1*Δ cells display features consistent with dysfunction in TOR signalling processes. **(A)** Cell viability upon nitrogen limitation was determined over periods of up to 72 hours in wild-type (WT) and *btn1*Δ cells, using the cell impermeable nucleotide stain propidium iodide to stain dead cells and calcofluor white to stain the total cell population. Cells were cultured in either MM or MM lacking a nitrogen source (NH_4_Cl). 500 cells were scored for viability per data set, and data shown is a mean (± SEM) of 3 independent experiments. **(B)** WT cells and *btn1*Δ cells were serially diluted from a log-phase culture (1 x 10^6^ cells/ml), and spotted onto plates containing either glucose or glycerol as a carbon source. Plates were then incubated at 30°C for 6 - 7 days to determine growth on fermentative and non-fermentative carbon sources. Images are representative of three independent experiments. **(C)** The morphological response of WT and *btn1*Δ cells to growth on glycerol was analysed following 6 hours in culture using a measure of cell elongation, on a scale of 0 to 1, where 0 represents a perfectly round cell (1 - (4 π area / perimeter^2^)). Data shown is a mean (± SEM) of 5 independent experiments. Statistical significance between each condition was determined using a one-way ANOVA with a Tukey’s multiple comparison post-test (** = P < 0.01). **(D)** Representative images of experiments as performed in (C) are shown. Scale bar represents 10 μm. **(E)** WT and *btn1*Δ cells were also serially diluted from a log-phase culture (1 x 10^6^ cells/ml) and spotted onto YES plates and YES plates containing 2M sorbitol. Plates were then incubated at 37°C for 3 - 4 days to determine the growth at high temperature and the influence of osmotic stabilisation. Images are representative of three independent experiments.

The interaction of *btn1* with core TORC components, and the link to surrounding signalling processes, provides compelling evidence for the importance of Tor signalling in cells lacking *btn1*. To validate this observation, we investigated *btn1*Δ cells for features consistent with Tor dysfunction. Repression of TORC1 activity is required to mount a correct response to nitrogen limitation 24. As loss of TORC1 activity was beneficial in cells lacking *btn1*, we hypothesised that these cells may display features consistent with dysregulation of TORC1 repression, and respond poorly to nitrogen limitation. To test this idea, we grew wild-type and *btn1*Δ cells in minimal media, and minimal media lacking a nitrogen source, over 72 h. Viability was monitored at 24 h intervals using propidium iodide to label dead cells and calcofluor white to label the total cell population (Fig. 2A). Cells lacking *btn1* displayed a consistently lower viability in media lacking nitrogen, falling to 81.5 ± 2.2% after 24 h compared to 98.7 ± 0.27% in minimal media containing nitrogen (P < 0.0015, unpaired t test), and remaining lower throughout the time course. Wild-type cells displayed no change in viability when cultured in media lacking nitrogen.

TORC1 repression and TORC2 signalling is also required to mount a response to glucose limitation 25,26. Cells that are defective in TORC2 function do not respond appropriately to glucose limitation by reducing their cell length 26. This is a relatively uncommon phenotype, with only *tor1* mutants and mutants in the Ca^2+^/calmodulin-dependent-like gene *ssp1 *known to display this phenotype 26,27. To test the ability of *btn1*Δ cells to adapt to conditions of limited glucose, we assessed both cell growth and the morphological response of these cells when grown with an alternative carbon source (glycerol). Cells lacking *btn1* displayed a clear growth defect under these conditions (Fig. 2B). Wild-type and *btn1*Δ cells grown for 6 h in media containing either glucose or glycerol as a carbon source were stained with calcofluor white to visualise the cell wall. Cell elongation was determined using a measure of cell circularity and given a score of between 0 and 1, where 0 represents a perfectly round cell (Fig. 2C and D). Wild-type cells displayed a significant reduction in cell elongation upon growth in glycerol (0.25 ± 0.01 to 0.16 ± 0.01, P ≤ 0.01). Cells lacking *btn1,* however, displayed no significant change (0.32 ± 0.02 to 0.31 ± 0.02), consistent with a defect in their response to glucose limitation that is most likely linked to Tor1 function.

Lastly, TORC2 and the connected CWI pathway are also required for resistance to high temperature 26. This temperature sensitivity of TORC2 and CWI pathway components is osmoremedial, i.e. it can be rescued by hypertonic growth media 28. We confirmed the temperature sensitivity of *btn1*Δ cells, and that it can be rescued with 2M sorbitol (Fig. 2E), consistent with previous observations 16.

These data together indicate that cells lacking *btn1* display many features consistent with defects in Tor signalling and function. Further, ectopic expression of *btn1* was able to rescue all these aspects of the mutant phenotype (Fig. S1A - D). Combined with the SGA results, these results suggest that Tor signalling is critical to the defects observed in *btn1*Δ cells.

### TORC2 and CWI pathway: modulating distinct signalling nodes elicit different levels of correction in *btn1*Δ cells

**Figure 3:**
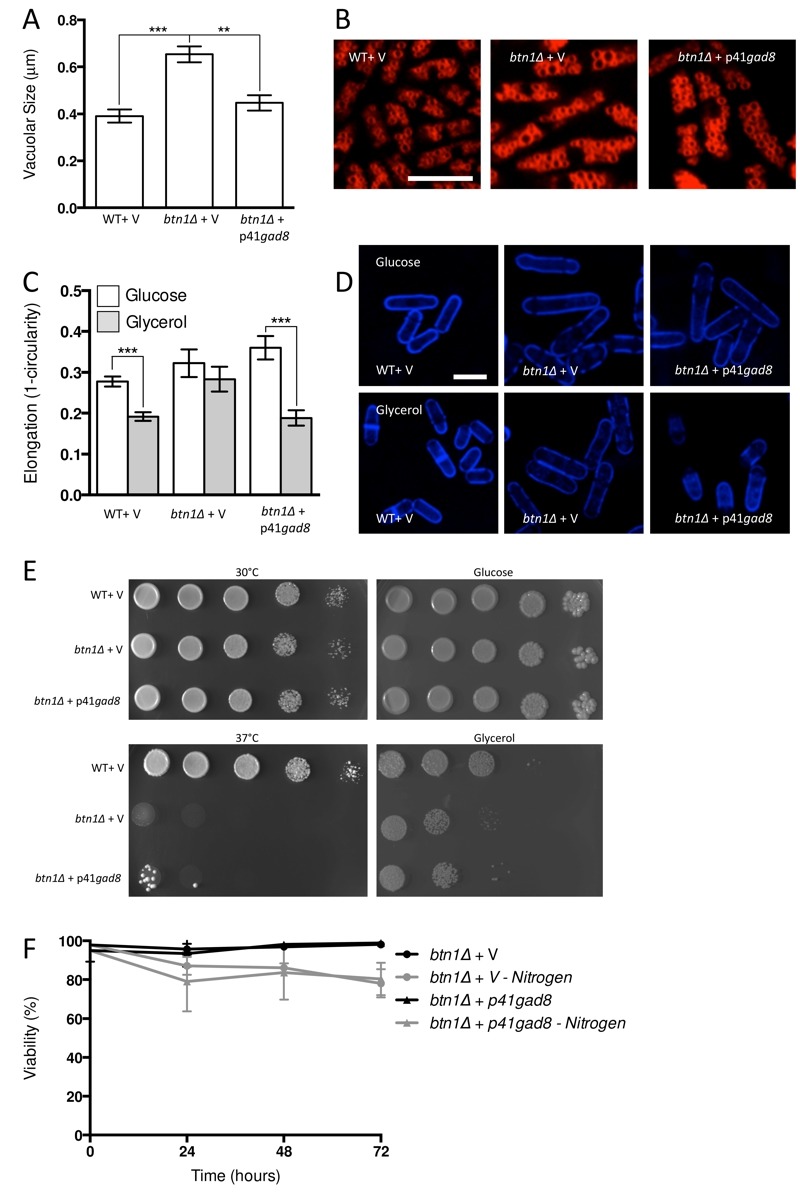
FIGURE 3: Increasing TORC2-dependent signalling rescues aspects of *btn1*Δ phenotype. **(A) **Cells lacking *btn1* were transformed with an expression vector containing *gad8. *Vacuole size was measured in these cells, in addition to WT and *btn1*Δ cells containing empty vector, following staining with the vital dye FM4-64. The diameter of 300 vacuoles was determined per data set, and data shown is a mean (± SEM) of 4 independent experiments. Statistical analysis was performed using a one-way ANOVA with a Tukey’s multiple comparison post-test (** = P < 0.01, *** = P < 0.001). **(B)** Representative images of experiments as performed in (A) are shown. Scale bar represents 10 μm. **(C)** The morphological response of these populations to growth on glycerol was analysed following 6 hours in culture using a measure of cell elongation, on a scale of 0 to 1, where 0 represents a perfectly round cell (1 - (4 π area / perimeter^2^)). Data shown is a mean (± SEM) of 5 independent experiments. Statistical significance between each condition was determined using a one-way ANOVA with a Tukey’s multiple comparison post-test (*** = P < 0.001). **(D)** Representative images of experiments as performed in (C) are shown. Scale bar represents 10 μm. **(E)** These cells were serially diluted from a log-phase culture (1 x 10^6^ cells/ml) and spotted onto YES plates. Plates were then incubated at 30°C or 37°C for 3 - 4 days to determine growth at high temperature. They were also spotted onto plates containing either glucose or glycerol as a carbon source. Plates were then incubated at 30°C for 6 - 7 days to determine growth under non-fermentative conditions. Images are representative of three independent experiments. **(F) **Viability upon nitrogen limitation was determined over periods of up to 72 hours in these cell populations, using propidium iodide to stain dead cells and calcofluor white to stain all cells. Cells were cultured in either MM or MM lacking a nitrogen source (NH_4_Cl). 500 cells were scored for viability per data set, and data shown is a mean (± SEM) of 3 independent experiments.

Given the negative genetic interaction between *btn1* and *tor1*, increasing TORC2 activity could alleviate aspects of the *btn1*Δ phenotype. We initially explored this approach through overexpression of the downstream kinase Gad8, which is a multicopy suppressor of aspects of the loss of TORC2 29. A key phenotype in cells lacking *btn1*, and one that is strongly linked to the phenotype of CLN3 disease, is a change in vacuole homeostasis 12. One of the clearest manifestations of this feature in *btn1*Δ cells is an increase in vacuole size 12. We investigated vacuole size in wild-type and *btn1*Δ cells containing empty vector alone (as a control), as well as *btn1*Δ cells expressing *gad8* from the *nmt1* promoter of the pREP41 plasmid (Fig. 3A and B). Consistent with previous reports, *btn1*Δ cells exhibited significantly larger vacuoles than wild-type cells (0.65 ± 0.03 μm compared to 0.39 ± 0.01 μm, P ≤ 0.001). The overexpression of *gad8* in *btn1*Δ cells significantly rescued vacuole size (0.40 ± 0.03 μm) (P ≤ 0.01), restoring them to near that of wild-type. These data provide the first indication that increasing TORC2 function significantly rescues a disease-relevant phenotype of *btn1*Δ cells.

As discussed above, the ability of cells to respond to glucose-limitation with a change in cell size requires TORC2 function 26. To investigate whether the inability of *btn1*Δ cells to undergo this morphological response can be rescued by increasing TORC2 pathway activity, we assessed the elongation of *btn1*Δ cells overexpressing *gad8 *following growth for 6 hours in media containing either glucose or glycerol as a carbon source (Fig. 3C and D). Wild-type and *btn1*Δ cells containing empty vector alone displayed comparable responses to cells without vector (WT glucose - 0.28 ± 0.01, WT glycerol - 0.19 ± 0.01, P ≤ 0.001; *btn1*Δ glucose - 0.32 ± 0.02, *btn1*Δ glycerol - 0.28 ± 0.01, ns). The overexpression of *gad8* was able to significantly rescue this morphological response, despite these cells being elongated under growth in glucose (glucose - 0.36 ± 0.01, glycerol - 0.19 ± 0.01, P ≤ 0.001).

Despite this rescue, expression of *gad8* was unable to significantly rescue the growth of *btn1*Δ cells on glycerol or at high temperature, suggesting that the ability of these cells to adapt to heat and nutrient stress is still impaired (Fig. 3E). Similarly, expression of *gad8* was unable to rescue the viability of *btn1*Δ cells grown in the absence of a nitrogen source (Fig. 3F).

Given that the phenotypes not rescued by increasing TORC2 activity were linked to stress and nutrient adaptation, we reasoned that modulating processes more closely linked to the CWI pathway might be more effective in correcting these phenotypes. These processes seem particularly relevant in this instance, as this pathway is known to respond to both heat and glucose limitation. In particular, mutants of the *pmk1* MAP kinase, like *btn1*Δ mutants, display a partial growth defect on glycerol. Further, this pathway is required for proper activation of Sty1 (the MAP kinase of the SAPK pathway) under glucose-limitation, integrating these processes with further identified *btn1* interactors 30.

**Figure 4:**
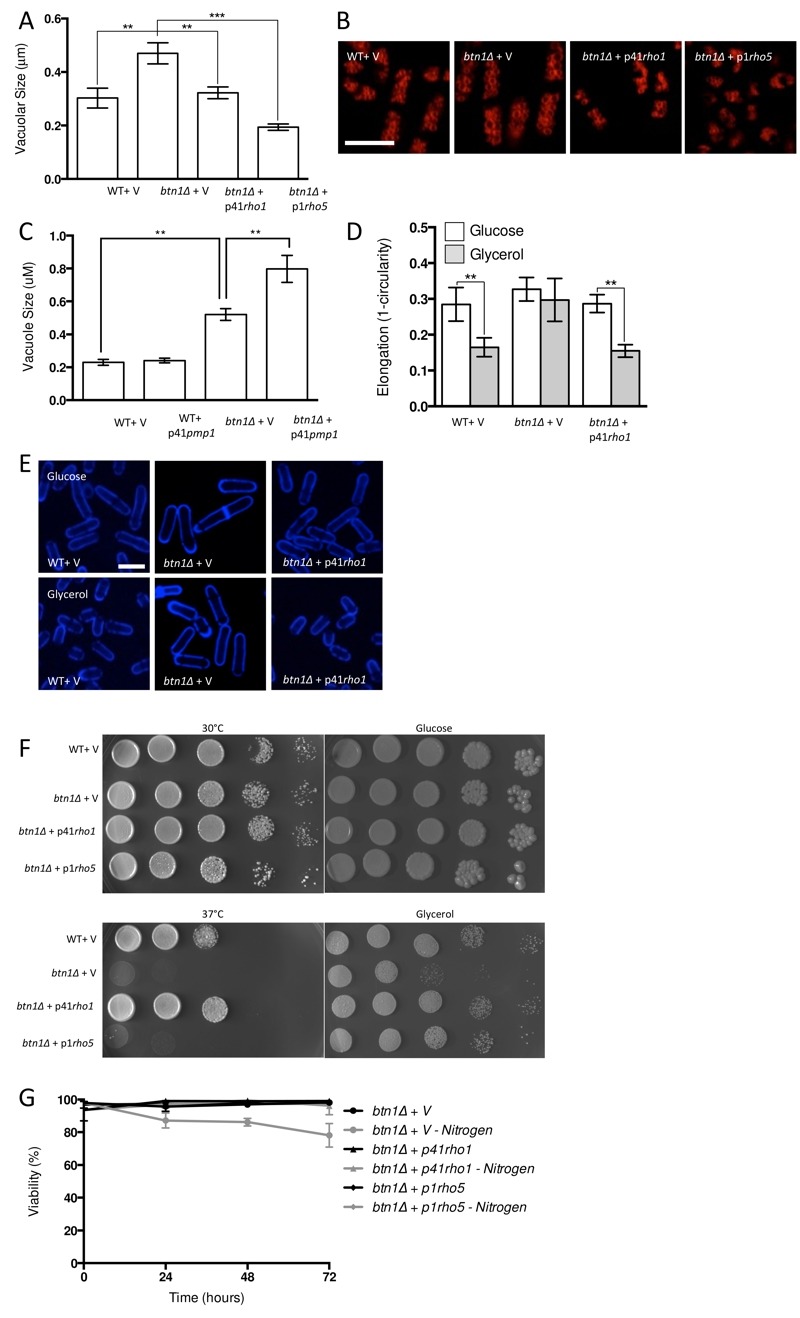
FIGURE 4: Overexpression of Rho rescues all aspects of *btn1*Δ phenotype. **(A) **Cells lacking *btn1* were transformed with an expression vector containing *rho1 *or* rho5. *Vacuole size was measured in these cells, in addition to wild-type (WT) and *btn1*Δ cells containing empty vector, following staining with the vital dye FM4-64. The diameter of 300 vacuoles was measured per data set, and data shown is a mean (±SEM) of 4 independent experiments. Statistical analysis was performed using a one-way ANOVA with a Tukey’s multiple comparison post-test (** = P < 0.01, *** = P < 0.001). **(B)** Representative images are shown in lower panels. Scale bar represents 10 μm. **(C) **WT and cells lacking *btn1* were transformed with an expression vector containing *pmp1. *Vacuoles were stained and measured as for (A). Statistical analysis was performed using a one-way ANOVA with a Tukey’s multiple comparison post-test (** = P < 0.01). **(D)** The morphological response of WT and *btn1*Δ cells containing empty vector as well as *btn1*Δ cells overexpressing *rho1* to growth on glycerol was analysed following 6 hours in culture using a measure of cell elongation, on a scale of 0 to 1, where 0 represents a perfectly round cell (1 - (4 π area / perimeter^2^)). Data shown is a mean (±SEM) of 5 independent experiments. Statistical significance between each condition was determined using a one-way ANOVA with a Tukey’s multiple comparison post-test (** = P < 0.01). **(E)** Representative images of experiments as performed in (D) are shown. Scale bar represents 10 μm. **(F)** Wild-type and *btn1*Δ cells containing empty vector as well as *btn1*Δ cells overexpressing *rho1 *and* rho5 *were serially diluted from a log-phase culture (1 x 10^6^ cells/ml) and spotted onto YES plates. Plates were then incubated at 30°C or 37°C for 3-4 days to determine growth at high temperature. They were also spotted onto plates containing either glucose or glycerol as a carbon source. Plates were then incubated at 30°C for 6-7 days to determine growth under non-fermentative conditions. Images are representative of three independent experiments. **(G) **Viability upon nitrogen limitation was determined over periods of up to 72 hours in these cell populations, using propidium iodide to stain dead cells and calcofluor white to stain all cells. Cells were cultured in either MM or MM lacking a nitrogen source (NH_4_Cl). 500 cells were scored for viability per data set, and data shown is a mean (±SEM) of 3 independent experiments.

To increase CWI activity, we chose to increase Rho GTPase levels in these cells, which represent a key hub in CWI pathway regulation, by overexpressing the *btn1* interactor *rho5* and its essential paralogue *rho1*. Overexpression of *rho1* significantly decreased vacuole size in *btn1*Δ cells (P ≤ 0.001) to near that of wild-type cells containing empty vector alone (WT - 0.30 ± 0.02 μm, *btn1*Δ* -* 0.47 ± 0.04 μm, *btn1*Δ with p41*rho1* - 0.32 ± 0.01 μm) (Fig. 4A and B). The overexpression of *rho5* led to an over-correction of this phenotype (0.19 ± 0.01 μm), producing vacuoles significantly smaller than those of *btn1*Δ cells (P ≤ 0.001) and wild-type cells containing vector alone (P ≤ 0.05). Consistent with these observations, over-expression of *pmp1*, which inhibits the activity of *pmk1 *31, caused an increase in vacuole size in *btn1*Δ cells (*btn1*Δ with p41*pmp1* - 0.80 ± 0.08 μm vs *btn1*Δ - 0.52 ± 0.04 μm, P ≤ 0.01) but not in wild-type cells (WT with p41*pmp1* - 0.24 ± 0.01 μm vs WT - 0.23 ± 0.02, ns) (Fig. 4C). Such data indicate that increasing CWI pathway activity is able to correct the vacuole defect of *btn1*Δ cells, and directly inhibiting this pathway exacerbates the vacuole defect. The over-correction by *rho5* could indicate a strong dose-dependence in this particular rescue. Indeed, previous work has demonstrated such dose dependence in the relationship between *btn1* and vacuole size 12.

We next investigated whether the Rho GTPases were able to rescue the morphological response of these cells to glycerol, as seen with *gad8*. We looked just at *rho1* in this case, as *rho5* overexpression has a pronounced effect on cell shape and septation 32, making morphological comparisons difficult (Fig. S2). Overexpression of *rho1* elicited a rescue of this response, with cells displaying a significant reduction in cell elongation when grown in glycerol containing media (0.28 ± 0.02 to 0.16 ± 0.01, P ≤ 0.01) (Fig. 4D and E).

Further to a rescue of the morphological response to glycerol, both *rho1* and *rho5* were able to correct the growth defect of *btn1*Δ cells on glycerol as a carbon source. In addition, *rho1* was able to rescue the temperature sensitivity phenotype of these cells (Fig. 4F). The overexpression of *rho5* did not correct this phenotype, although, given the link between heat-sensitivity and septation defects in *btn1*Δ cells 15 and the enhanced septation defect of cells overexpressing *rho5 *(Fig. S2), this is not wholly unexpected.

In addition to these phenotypes, overexpression of *rho1* and *rho5* rescued the viability of *btn1*Δ cells when grown in the absence of a nitrogen source, with viability remaining higher than 95% throughout the course of the experiment (Fig. 4G). These data provide evidence for a profound positive interaction between CWI pathway signalling and *btn1*, indicating that such processes are intimately linked with the defects that occur in *btn1*Δ cells.

### Reducing TORC1 activity corrects defects in *btn1*Δ cells

TORC1 activity is a key signal of a favourable environment, promoting proliferation and suppressing stress-responsive processes 24. As a consequence, it is antagonistic to many of the processes explored in this study that improved the *btn1*Δ phenotype. TORC1 represses TORC2 function 23, and negatively regulates Sty1 33, which displays extensive cross-talk with the CWI pathway 30. This relationship is highlighted by the fact that the core TORC1 component gene *tco89* is a positive interactor of *btn1*.

**Figure 5:**
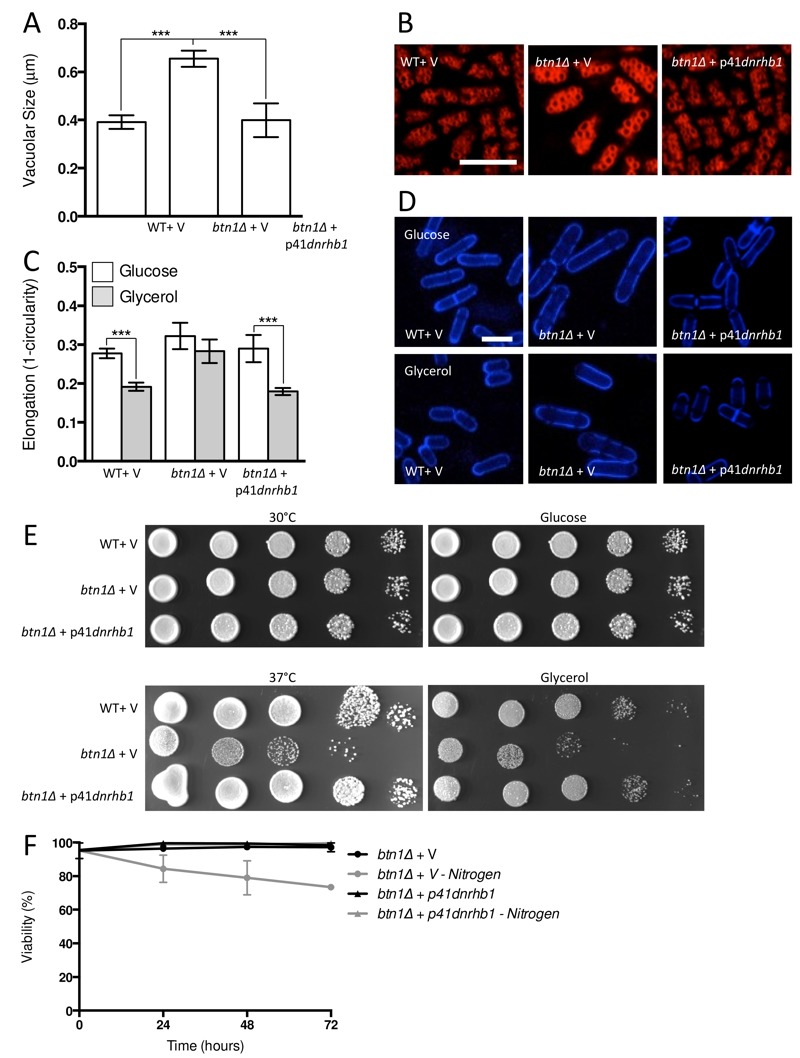
FIGURE 5: Inhibiting TORC1 function rescues the defects in *btn1*Δ cells. **(A) **Cells lacking *btn1* were transformed with an expression vector containing a dominant-negative form of *rhb1* (*dnrhb1*)*. *Vacuole size was measured in these cells, in addition to wild-type (WT) and *btn1*Δ cells containing empty vector, following staining with the vital dye FM4-64. The diameter of 300 vacuoles was measured per data set, and data shown is a mean (± SEM) of 4 independent experiments. Statistical analysis was performed using a one-way ANOVA with a Tukey’s multiple comparison post-test (*** = P < 0.001). **(B)** Representative images of experiments as performed in (A) shown. Scale bar represents 10 μm. **(C)** The morphological response of these populations to growth on glycerol was analysed following 6 hours in culture using a measure of cell elongation, on a scale of 0 to 1, where 0 represents a perfectly round cell (1 - (4 π area / perimeter^2^)). Data shown is a mean (± SEM) of 5 independent experiments. Statistical significance between each condition was determined using a one-way ANOVA with a Tukey’s multiple comparison post-test (*** = P < 0.001). **(D)** Representative images of experiments as performed in (C) shown. Scale bar represents 10 μm. **(E)** These cells were serially diluted from a log-phase culture (1 x 10^6^ cells/ml) and spotted onto YES plates. Plates were then incubated at 30°C or 37°C for 3-4 days to determine growth at high temperature. They were also spotted onto plates containing either glucose or glycerol as a carbon source. Plates were then incubated at 30°C for 6-7 days to determine growth under non-fermentative conditions. Images are representative of three independent experiments. **(F) **Viability upon nitrogen limitation was determined over periods of up to 72 hours in these cell populations, using propidium iodide to stain dead cells and calcofluor white to stain all cells. Cells were cultured in either MM or MM lacking a nitrogen source (NH_4_Cl). 500 cells were scored for viability per data set, and data shown is a mean (± SEM) of 3 independent experiments. Scale bar represents 10 μm.

In order to test whether repression of TORC1 activity could rescue the phenotypes of *btn1*Δ cells, we first overexpressed a dominant-negative form of the upstream activator of TORC1, Rhb1 (*rhb1^D60K^*) 34. Upon overexpression of dominant-negative *rhb1* (*dnrhb1*) in *btn1*Δ cells, we observed a significant reduction (P ≤ 0.001) in vacuole size compared to *btn1*Δ cells containing vector alone (*btn1*Δ with p41*dnrhb1* - 0.40 ± 0.04 μm, *btn1*Δ with empty vector - 0.65 ± 0.03 μm) (Fig. 5A and B). This result was comparable to the vacuole size of wild-type cells (0.39 ± 0.01 μm), indicating a rescue of vacuole morphology upon repression of TORC1 activity.

We next investigated the ability of TORC1 repression to rescue the nutrient-sensing defects of cells lacking *btn1*. The overexpression of *dnrhb1* led to a significant rescue of the morphological response of these cells to glucose-limitation, following 6 hours growth in glycerol as a carbon source (Fig. 5C and D). Cell elongation fell from 0.29 ± 0.02 to 0.18 ± 0.01 when grown in glycerol as opposed to glucose, a response comparable to that observed in wild-type cells (glucose - 0.28 ± 0.01, glycerol - 0.19 ± 0.01). This also corresponded to a rescue of the growth defect of these cells on glycerol (Fig. 5E). Further to the glycerol growth defect, *dnrhb1* overexpression also rescued the heat sensitivity of *btn1*Δ cells (Fig. 5E). Finally, this construct was also able to elicit a complete rescue of viability under nitrogen limiting conditions, with viability remaining above 95% throughout the course of the experiment (Fig. 5F).

**Figure 6:**
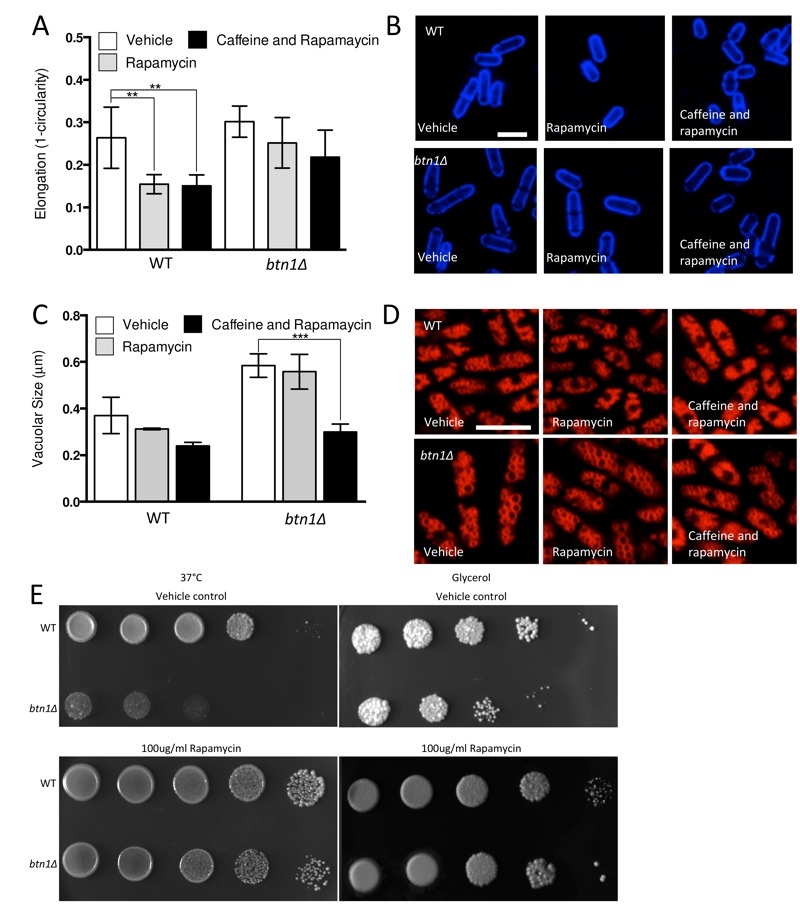
FIGURE 6: Pharmacological inhibition of TORC1 function replicates rescue by overexpression of dominant-negative *rhb1.* **(A) **The morphological response of wild-type (WT) and *btn1*Δ cells to the TORC1 antagonists rapamycin (100 μg/ml) and caffeine and rapamycin combined (10 mM and 100 μg/ml respectively) was analysed following 6 hours in culture using a measure of cell elongation, on a scale of 0 to 1, where 0 represents a perfectly round cell (1 - (4 π area / perimeter^2^)). Data shown is a mean (± SEM) of 6 independent experiments. Statistical significance between each condition was determined using a one-way ANOVA with a Tukey’s multiple comparison post-test (** = P < 0.01). **(B)** Representative images of experiments as performed in (A) are shown. Scale bar represents 10 μm. **(C)** Wild-type and *btn1*Δ cells were grown for 6 hours in MM alone or with the TORC1 antagonists rapamycin (100 μg/ml) and caffeine and rapamycin (10 mM and 100 μg/ml respectively). Vacuole size was then measured in these cells following staining with the vital dye FM4-64. The diameter of 300 vacuoles was measured per data set, and data shown is a mean (± SEM) of 4 independent experiments. Statistical analysis was performed using a one-way ANOVA with a Tukey’s multiple comparison post-test (*** = P < 0.001). **(D)** Representative images of experiments as performed in (C) are shown. Scale bar represents 10 μm. **(E)** WT and *btn1*Δ cells were serially diluted from a log-phase culture (1 x 10^6^ cells/ml) and spotted onto YES plates either containing or lacking rapamycin (100 μg/ml). Plates were then incubated at 37°C for 3-4 days to determine growth at high temperature. They were also spotted onto plates containing glycerol as a carbon source either containing or lacking rapamycin (100 μg/ml). Plates were then incubated at 30°C for 6-7 days to determine growth under non-fermentative conditions. Images are representative of three independent experiments.

Given the substantial rescue of *btn1*Δ cells by *dnrhb1*-mediated activation of TORC1, we wanted to confirm the interaction between *btn1* and TORC1 by another means. We chose to use two pharmacological inhibitors of TORC1, rapamycin (allosteric) and caffeine (competitive inhibitor of ATP binding) 35. TORC1 antagonism leads to cell rounding, a response that mimics the morphological change ordinarily exhibited by wild-type cells in response to nitrogen and amino acid starvation. Therefore, we examined the effect of TORC1 antagonism on cell rounding 36 and on vacuole size, heat sensitivity, and growth in glycerol. As expected, wild-type cells displayed a significant reduction in cell elongation upon rapamycin treatment for 6 hours (0.26 ± 0.02 to 0.15 ± 0.01, P ≤ 0.01) (Fig. 6A and B). This reduction in cell elongation did not change further upon exposure to both rapamycin and caffeine 35. Cells lacking *btn1,* however, did not respond to rapamycin, displaying only a slight morphological change (0.30 ± 0.01 to 0.25 ± 0.02) that was slightly enhanced by the addition of caffeine and rapamycin, but again not significantly (0.22 ± 0.03) (Fig. 6A and B). The addition of caffeine and rapamycin to wild-type cells did not significantly affect vacuole size (vehicle - 0.37 ± 0.05 μm, rapamycin - 0.31 ± 0.03, caffeine and rapamycin 0.24 ± 0.01) (Fig. 6C and D). Rapamycin alone had no effect on the vacuole size of cells lacking *btn1*, but, in contrast, a combination of caffeine and rapamycin significantly (P ≤ 0.001) reduced vacuole size (vehicle - 0.58 ± 0.03 μm, rapamycin - 0.55 ± 0.04, caffeine and rapamycin 0.30 ± 0.02) (Fig. 6C and D). Further, treatment with rapamycin alone elicited a rescue of both the heat sensitivity defect and glycerol growth defect of cells lacking *btn1 *(Fig. 6E). The ability of these pharmacological treatments to rescue the defects in vacuole size, heat sensitivity and growth in glycerol of *btn1*Δ cells appears to be closely linked to their ability to target TORC1 signalling itself, as opposed to changes in the activity of the connected SAPK pathway, as expression of a constitutively active *wis1* mutant (*wis1DD*) 37 did not rescue these phenotypes (Fig S3A - C). These data confirm that activation of the TORC1 pathway pharmacologically in *btn1*Δ cells rescues most of the phenotypes arising from loss of *btn1 *function. However, the pharmacological inhibition of the TORC1 pathway is not completely equivalent to the inhibition of the TORC1 pathway using *dnrhb1* in *btn1*Δ cells.

## DISCUSSION

**Figure 7:**
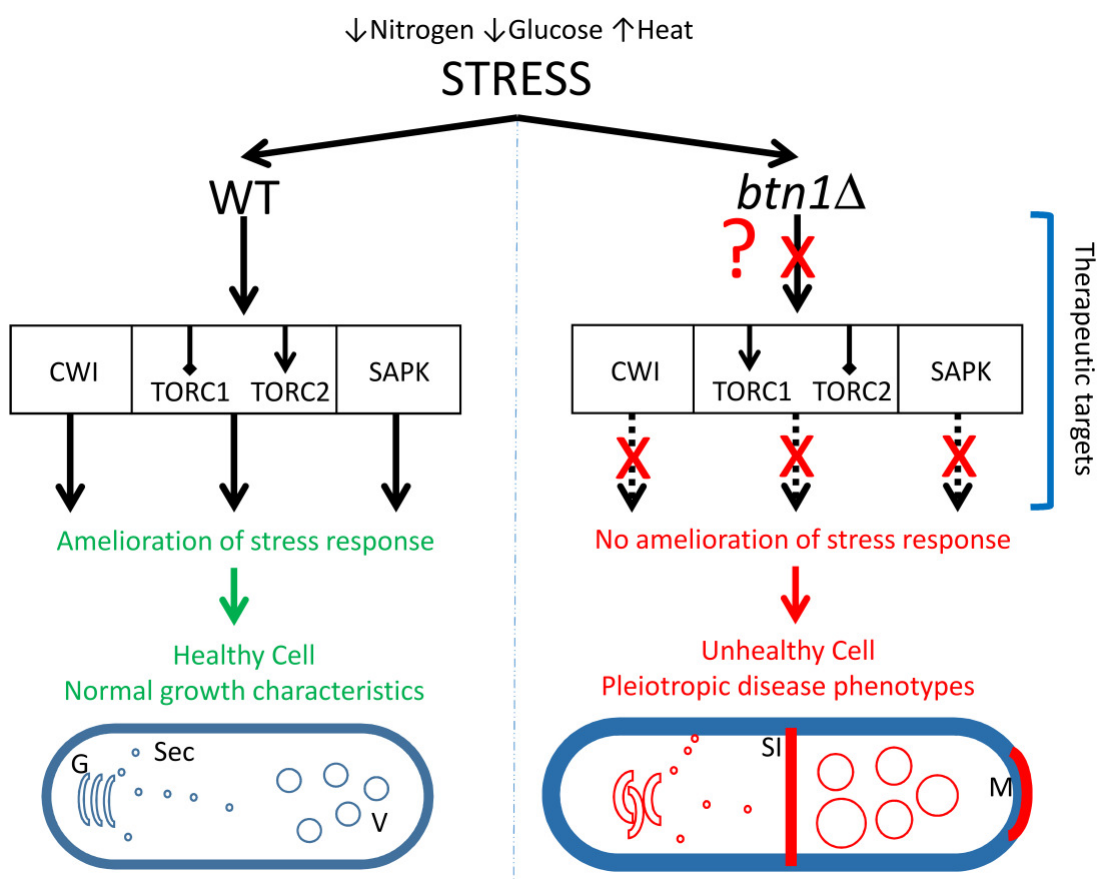
FIGURE 7: Diagrammatic summary of the effect of loss of *btn1* on the response to stress. The left panel shows the response of healthy cells to stresses applied in this study, such as low nitrogen or glucose or raised temperature. These cells are able to respond via interconnected signaling pathways such as CWI, TORC and SAPK. Activation of these pathways allows the cell to respond to the effects of stress and restore cell homeostasis and integrity. In contrast, cells lacking *btn1* are unable to respond to the applied stresses (represented by ‘?’ and ‘X’) and undergo a variety of previously reported responses that include an increase in vacuole size, swollen and disorganized Golgi and mis-trafficking of vacuole enzymes such as carboxypeptidase Y, increased septation index and longer cell cycle and cell length, monopolar growth, defective cell wall, absence of cell rounding (in response to low glucose). Genetic or pharmacological activation of the three signalling pathway provides partial or full rescue of these pleiotropic phenotypes of *btn1*Δ cells. G, Golgi; Sec, secretory vesicles; V, vacuole; SI, division septum; M, monopolar growth.

In this study, SGA analysis was used as an unbiased approach to identify the genetic interactors of *btn1, *and place *btn1* into its biological context within the whole cell. This approach highlighted a key role for the Tor kinase complexes, TORC1 and TORC2, in the dysfunctional changes that occur in fission yeast lacking *btn1*, as one third of the interactions connected directly with pathways in which these complexes are active. Further, we have shown that *btn1*Δ cells display defects in their stress response to nitrogen and glucose limitation, in addition to osmoremedial heat sensitivity, consistent with TORC1 and TORC2 dysfunction in these cells (Fig. 7).

Specifically, the SGA identified negative interactions in *tor1*, the core kinase component of TORC2, multiple components of the stress-activated protein kinase (SAPK) pathway, and a component of the linked CWI pathway. In addition, *tco89*, encoding a core component of TORC1, which antagonises both the SAPK cascade and TORC2 signalling, was identified as a positive genetic interactor of *btn1*. *btn1* was also found to interact negatively with a number of genes that display stress-responsive expression.

TORC1 negatively regulates a number of processes associated with cellular catabolism and adaptation to stress 24. One of these roles is in the repression of autophagy. Autophagy similarly appears important to the fitness of *btn1*Δ cells, as *atg14* and *atg12* were identified as negative interactors of *btn1*, and both are required for autophagosome formation 38. In support of this work, and the use of yeast as a model, defective autophagy has previously been linked to CLN3 disease 39. In repressing stress-responsive processes, TORC1 is known to negatively regulate *sty1*, the mitogen-activated protein (MAP) kinase and component of the stress-activated protein kinase (SAPK) cascade 33. Both *wis4* and *wis1*, encoding the MAP kinase kinase kinase and MAP kinase kinase of the SAPK pathway, respectively, interact negatively with *btn1*, as does the transcription factor gene *pap1*, which is a downstream target of *sty1* 40. Furthermore, *btn1* interacts negatively with a number of genes that display stress-responsive expression.

TORC2 is a positive regulator of stress-responsive processes 29, and is also involved in cytoskeletal organization, vacuolar morphology and glucose sensing 22. In addition to a negative interaction with the gene encoding the Tor kinase component of TORC2 (*tor1*), *btn1* also negatively interacts with genes encoding components of the connected CWI MAP kinase cascade. The relationship between TORC2 and the CWI pathway is complex. TORC2 is required for the response to cell wall stressors 41, and is a direct regulator of the CWI pathway through the activation of the Rho guanine-nucleotide exchange factor (GEF) ROM2 in budding yeast 42. In fission yeast, it has recently been shown that Tor1, acting as part of TORC2, enhances Pck2 synthesis to activate the CWI under both cell wall stress and glucose limitation conditions 43.

Despite this positive relationship, recent work in fission yeast has indicated a negative regulation of TORC2 by *pmk1*, the MAP kinase component of the fission yeast CWI pathway, at least in certain conditions 25. In fission yeast both Rho1 and Rho2 are well established as regulators of the CWI pathway 44,45. Rho5 acts as a functional homologue of *rho1,* sharing the role of regulating cell wall homeostasis 46. More recently, *rho5* has also been shown to be an upstream regulator of the CWI 47. The regulation of this pathway is complex as Rho1 both positively 45 and negatively 48 regulates the downstream MAP kinase cascade. This potentially intricate relationship is reflected in the interactors of *btn1* that fall within this pathway, that exhibit both positive and negative effects upon signalling. *btn1* exhibits a negative interaction with the *rho1* homologue gene *rho5*, and a number of genes involved in downstream cytoskeletal and polarity components. Despite this, the Rho GEF gene *rgf1*, which is a positive regulator of the CWI pathway 49, is a positive interactor of *btn1* whereas *rdi1* (encoding a GDP dissociation factor) is a negative interactor. This multifaceted pattern of interactions is consistent with current understanding of this pathway, given the complex interactions between these signalling nodes.

Although a process that maintains CWI might seem fungal-specific, and not relevant to disease in a higher eukaryote, previous work in budding yeast revealed similar dysfunctional processes in cells overexpressing α-synuclein as a model for Parkinson’s disease, suggesting that there is a mammalian counterpart to the CWI pathway in yeast. Such observations are of specific relevance to CLN3 disease, as α-synuclein is also upregulated in this condition 9. RHO1 signalling and the CWI pathway are disrupted by overexpression of α-synuclein 50. Further, it was demonstrated that similar changes in downstream kinase cascades could be observed in mammalian cells overexpressing α-synuclein. Affected kinases included c-Jun, which has previously been linked to CLN3 disease, and is part of a pathway closely related to the SAPK pathway in yeast 51. This is not the only instance in which Rho signalling has been linked to neurodegeneration. Leucine-rich repeat kinase 2 (LRRK2), mutations in which represent the most common cause of familial late-onset Parkinson’s disease, has been shown to bind Rac1, and to a lesser extent RhoA and Cdc42 52. All of these binding partners are members of the Rho GTPase family. The binding of LRRK2 to Rac1 led to its activation and relocalisation, and was lost when disease-causing mutations were introduced into LRRK2. Further, the overexpression of Rac1 was shown to be protective in these cells. Other studies have also demonstrated a protective effect of Rho signalling, focusing both on Rho GEFs 53 and the Rho GTPases themselves 54. Such studies highlight how processes as seemingly specialized as CWI in yeast can be highly informative of processes relevant to disease in higher order eukaryotes. Further, they demonstrate that Rho-dependent signalling could be a vital neuroprotective process in more common neurodegenerative disease, as well as being a key pathway in the neurodegenerative disease model presented in this study.

Importantly, the validation of these interactions during this work revealed interventions that produced a complete rescue of all phenotypes investigated in the *btn1*Δ strain, including phenotypes linked to TORC1 function, TORC2 function, CWI and vacuole homeostasis. Among the interactions investigated, increasing Rho1 levels, and thereby upregulating the main signalling node in the CWI pathway, or repressing TORC1 function (pharmacologically or genetically) led to complete rescues.

More notably, a neuroprotective role of TORC1 repression is already well established in a number of different systems 55. A protective effect of the TORC1 antagonist rapamycin has been demonstrated in models for Parkinson’s disease 57, Huntington’s disease 56, spinoceribellar ataxia type 3 58 and retinal degeneration mediated by mitochondrial dysfunction 59. In addition, Tor signalling is also elevated in Alzheimer’s disease models 60.

It is important to note, however, that targeting TORC1 in disease presents some technical challenges. Although in some studies an increase in lysosome function has been observed upon rapamycin treatment 61, other work indicates that rapamycin is an incomplete antagonist of TORC1 and a poor inducer of lysosomal functions 62, in concert with our observations. Another complication of targeting TORC1 in disease is the antagonistic role of TORC2. TORC2 signalling and the connected CWI pathway were both protective in our model for CLN3 disease. Further to the protective role of Rho signalling in a Parkinson’s disease model 50, TORC2/AKT signalling has also been shown to be neuroprotective in a separate study of Parkinson’s disease 63. As a result, general Tor antagonists, such as Torin1, which target both TORC1 and TORC2, do not produce the same positive outcomes that TORC1 specific antagonists are able to elicit 57. In addition, chronic rapamycin exposure also leads to a reduction in TORC2 activity. This fact could explain why rapamycin has been shown to be detrimental in certain studies, for example in one study using a fruit fly model for Alzheimer’s disease 64.

## Conclusion

The data presented here highlight a set of interconnected pathways that, when modulated correctly, produce a profound rescue of the dysfunctional changes observed in a yeast model for neurodegeneration caused by mutations in a single gene (Fig. 7). Links between CLN3 and the response to stress have previously been reported in other model organisms 51,65, supporting the relevance of this yeast work to human CLN3 disease. Importantly however, a link between CLN3 and Tor signalling specifically, as reported here, is a novel mechanistic and therapeutic insight into CLN3 disease, the most common paediatric neurodegenerative disease. Furthermore, the pathways identified display strong parallels with known protective pathways in more common dementias, providing not only strong biological plausibility for the importance of these interactions, but also an indication that common processes may be exploited in therapeutic development for seemingly disparate neurodegenerative diseases.

## MATERIALS AND METHODS

### Yeast strains and cell growth

The strains used in this study were the wild-type strains SP23 (*h-, ura4-D18, leu1-32, ade6-M210*) and SP38 (*h-, ura4-D18, leu1-32, ade6-210, his-*) and *btn1*Δ strains SP29 (*h-, btn1::NatMX, ura4-D18, leu1-32, ade6-210,*) and SP35 (*h-, btn1::NatMX, ura4-D18, leu1-32, ade6-210, his-*). SP29 and SP35 were generated using the long primer method described in 66, using pFA6a-*NatMX* as a template 67. Both strain backgrounds were used for SGA analysis (two isolates of each) to account for strain background differences, and SP23 and SP29 were used in all other analyses. Cell growth and manipulations were performed as described previously 68. Minimal media (MM) was used in all experiments, unless otherwise stated. In nitrogen starvation experiments NH_4_Cl was omitted. Glucose and glycerol media were made as described previously 69. Appropriate supplements were added to the media as required. All assays were performed using log-phase cultures (1 x 10^6^ cells/ml). The *dnrhb1* construct (pREP4X-*rhb1^D60K^*) was obtained from Dr. Elizabeth Henske 34, and the *gad8* construct (pREP41-*gad8*) from Dr. Ronit Weisman 70. pREP1-*rho5* was obtained from Dr. Kentaro Nakano 46, pREP41-*rho1* from Dr. Pilar Pérez 48 and pREP1-*wis1DD-3HA6His* from Dr. Kaz Shiozaki 71. The *GFP-btn1* construct used in this study was described previously (pREP41-*GFP-btn1*) 12. SP23 containing empty vector (pREP41) was used as a control in all experiments using these constructs.

### Synthetic genetic arrays (SGAs)

SGA analysis was performed as described in 20, using a Singer RoToR robot (*S. pombe* settings) and the Bioneer haploid library (v2.0) and analysed as described previously 72. A control SGA using *ade6::NatMX4* as a query was performed simultaneously. The *ade6*Δ mutant does not alter the fitness of the deletion collection, and arising double mutants are used to determine Bioneer strain fitness and for colony size normalisation. After germination, haploid double mutants were pinned to YES + G418 + Nat in a 1536 format. After three days growth at 30°C, agar plates were imaged using a MultiDoc-It Imaging System (UVP) and processed for analysis using the Gitter image analysis R package 73. Using Excel, the colony sizes produced by Gitter were normalized to the median colony size of each plate to account for within-plate positional growth effects and differential plate growth. These normalized values were used to establish the ratio of query *btn*1**Δ mutant size to control (*ade6:NatMX4*) colony size. Ratios from the four *btn*1**Δ isolates used for the screen were used to calculate a mean ratio. Using this mean value, colony-size ratio scores of < 0.8 were defined as negative interactions, and ratios of > 1.2 as positive interactions. Genetic interactions were scored from two independent biological experiments. Only interactions common to both replicates were considered robust enough for follow-up. Negative and positive interaction sets were tested using PANTHER 74.

### Microscopy

All images were obtained using a Leica SPE2 true scanning confocal microscope with 63 x 1.4 NA oil immersion objective. Images were recorded using Leica AF software. Calcofluor-white (Sigma-Aldrich) staining was performed as described previously 68. Calcofluor-white was used to allow cell segmentation for assays of cell morphology. For assays of cell viability, propidium iodide (Sigma-Aldrich) was added to the cells (15 μg/ml final concentration) at the same point as the calcofluor-white. Staining was performed in a volume of 1 ml, allowed to proceed at room temperature for 10 min, and the cells were washed twice in MM before visualisation. No cell fixation was applied. The vital dye FM4-64 (Molecular Probes) was used to visualise vacuolar morphology. The staining procedure has been described previously 12.

### Image analysis

All microscopy image analysis was performed using ImageJ software. For analysis of cell morphology, cells were segmented using the BOA plugin of the quantitative imaging of membrane proteins (Quimp) package 75. Cell elongation was then determined using the equation: (1 - (4 π area / perimeter^2^)). Thirty cells were counted per dataset.

### Statistical analysis

All statistical analysis was performed using Prism software, version 6.0C (Graphpad). In instances where only two samples were compared, significance was determined using an unpaired t test. In instances where multiple columns were compared, an ordinary one-way ANOVA was used with a Tukey’s multiple-comparison post-test. All error bars represent mean ± standard error of the mean (SEM). In all cases, the statistical test used, p-values and the number of samples analysed are highlighted in the figure legend for clarity.

## SUPPLEMENTAL MATERIAL

Click here for supplemental data file.

All supplemental data for this article are also available online at http://microbialcell.com/researcharticles/a-central-role-for-tor-signalling-in-a-yeast-model-for-juvenile-cln3-disease/.
