# Supplementary material for: A central role for TOR signalling in a yeast model for juvenile CLN3 disease
Source: Microb Cell. 2015 Nov 11;2(12):466–80. doi: 10.15698/mic2015.12.241 (PMC5354605; doi:10.15698/mic2015.12.241)
Supplement: Supplementary file 1 [file mic-02-466-s01.pdf]

A central role for TOR signalling in a yeast model for juvenile CLN3 disease

Michael E. Bond,[1] Rachel Brown,[1] Charalampos Rallis,[3,4] Jürg Bähler,[3,4] and Sara E. Mole[1,2,3]*

[1]MRC Laboratory for Molecular Cell Biology, [2]Institute of Child Health, [3]Department of Genetics, Evolution and Environment, and [4]Institute of Healthy Ageing, University College London, London WC1E 6BT, UK

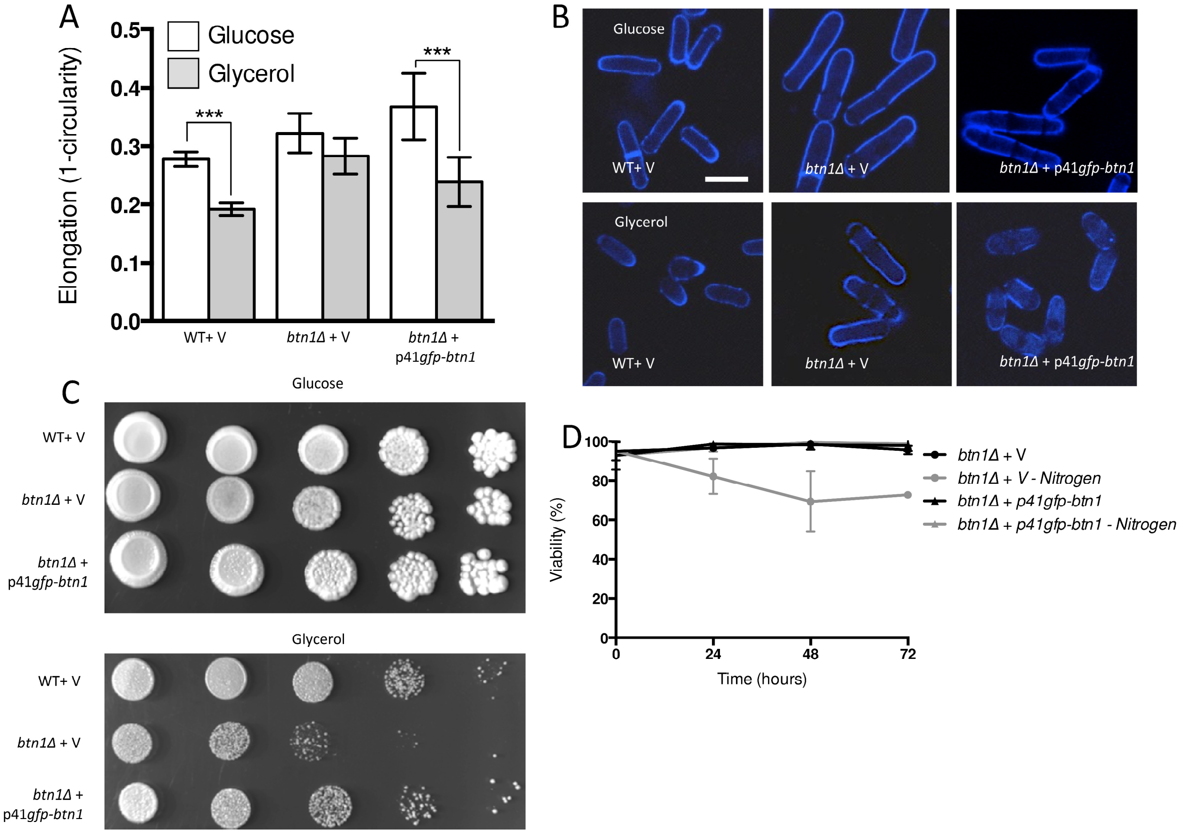

Figure S1. Overexpression of *btn1* rescues tor-related phenotypes in cells lacking *btn1*.

(A) Cells lacking *btn1* were transformed with an expression vector containing *gfp-btn1.* The morphological response of this population, in addition to wild-type and *btn1Δ* cells containing empty vector, to growth on glycerol (glucose-limitation) was analysed following 6 hours in culture using a measure of cell elongation, on a scale of 0 to 1, where 0 represents a perfectly round cell $(1 - (\frac{4\,\pi\,area}{perimeter^2}))$. Data shown is a mean (±SEM) of 5 independent experiments. Statistical significance between each condition was determined using a one-way ANOVA with a Tukey's multiple comparison post-test (*** = p<0.001). **(B)** Representative images of experiments performed as in (A) are shown. Scale bar represents 10 µm. **(C)** These cells were serially diluted from a log-phase culture ($1x10^6$ cells/ml) and spotted onto plates containing either glucose or glycerol as a carbon source. Plates were then incubated at 30°C for 6-7 days to determine growth under non-fermentative conditions. **(D)** Viability upon nitrogen limitation was determined over periods of up to 72 hours in these cell populations, using the cell impermeant nucleotide stain propidium iodide to stain dead cells and calcofluor white to stain the total cell population. Cells were cultured in either MM or MM lacking a nitrogen source (NH_4Cl). Data shown is a mean (±SEM) of 3 independent experiments.

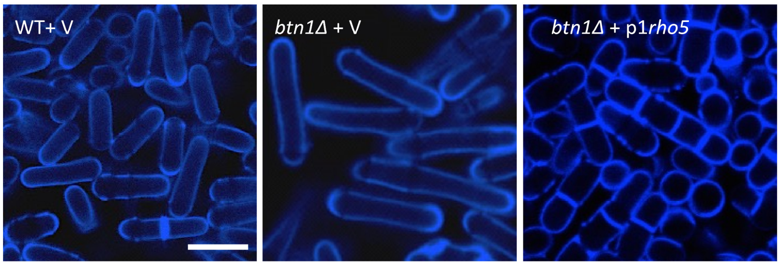

Figure S2. Overexpression of *rho5* leads to septation and morphology defects.

Cells lacking *btn1* were transformed with an expression vector containing *rho5.* These cells, in addition to wild-type and *btn1Δ* cells containing empty vector, were cultured for 24 hours, stained with calcofluor white to visualize the cell wall and then imaged to assess morphology and septation. Scale bar represents 10 µm.

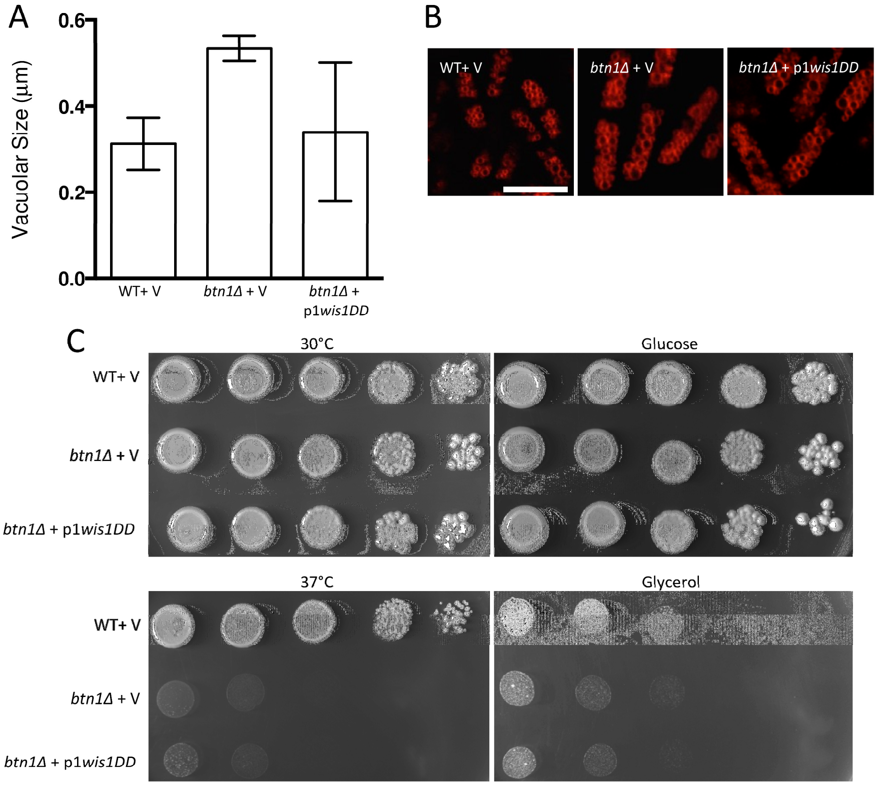

Figure S3. Activation of the SAPK pathway with overexpressing a constitutively active *wis1* (*wis1DD*) does not rescue *btn1Δ* phenotypes.

(A) Cells lacking *btn1* were transformed with an expression vector containing a constitutive form of *wis1* (*wis1DD*)*.* Vacuole size was measured in these cells, in addition to wild-type and *btn1* Δ cells containing empty vector, following staining with the vital dye FM4-64. Data shown is a mean (±SEM) of 4 independent experiments. Statistical analysis was performed using a one-way ANOVA with a Tukey's multiple comparison post-test. **(B)** Representative images of experiments performed as in (A) are shown. Scale bar represents 10 µm. **(C)** These cells were serially diluted from a log-phase culture ($1x10^{6}$ cells/ml) and spotted onto YES plates. Plates were then incubated at 30°C or 37°C for 3-4 days to determine growth at high temperature. They were also spotted onto plates containing either glucose or glycerol as a carbon source. Plates were then incubated at 30°C for 6-7 days to determine growth under non-fermentative conditions.

Table S1A: Negative Interactors of *btn1*

ID	Name	Description
SPAC1002.07c	ats1	N-acetyltransferase Ats1 (predicted)
SPAC11G7.01	SPAC11G7.01	serine-rich Schizosaccharomyces specific protein
SPAC1250.04c	atl1	alkyltransferase-like protein Atl1
SPAC1296.01c	SPAC1296.01c	phosphoacetylglucosamine mutase (predicted)
SPAC1399.04c	SPAC1399.04c	uracil phosphoribosyltransferase (predicted)
SPAC13D6.01	pof14	F-box protein Pof14
SPAC13D6.02c	byr3	translational activator, zf-CCHC type zinc finger protein (predicted)
SPAC13D6.04c	btb3	substrate adaptor for cullin 3 ubiquitin ligase Btb3
SPAC13F5.01c	msh1	mitochondrial MutS protein Msh1 (predicted)
SPAC13F5.03c	gld1	mitochondrial glycerol dehydrogenase Gld1
SPAC13F5.04c	vta1	Vps20 associated protein Vta1 (predicted)
SPAC13F5.05	SPAC13F5.05	thioredoxin family protein (predicted)
SPAC13F5.07c	SPAC13F5.07c	zf PARP type zinc finger protein
SPAC13G7.02c	ssa1	heat shock protein Ssa1 (predicted)
SPAC13G7.09c	SPAC13G7.09c	conserved fungal protein
SPAC140.03	arb1	argonaute inhibitor protein 1
SPAC15A10.06	SPAC15A10.06	CPA1 sodium ion/proton antiporter (predicted)
SPAC1783.01	SPAC1783.01	methionine synthase reductase (predicted)
SPAC1783.06c	atg12	autophagy associated ubiquitin-like modifier Atg12
SPAC1783.07c	pap1	transcription factor Pap1/Caf3
SPAC17A5.09c	glc9	protein phosphatase regulatory subunit Glc9 (predicted)
SPAC17A5.16	ftp105	Ubp5 interacting protein Ftp105
SPAC17A5.18c	rec25	meiotic recombination protein Rec25
SPAC17G6.17	pof8	F-box protein Pof8
SPAC17G8.10c	dma1	mitotic spindle checkpoint ubiquitin ligase Dma1
SPAC17H9.03c	rdl1	RAD51D-like protein 1
SPAC17H9.04c	SPAC17H9.04c	RNA-binding protein
SPAC17H9.06c	SPAC17H9.06c	conserved eukaryotic protein
SPAC17H9.11	gmf1	cofilin/tropomyosin family Glia Maturation Factor homolog Gmf1
SPAC17H9.12c	SPAC17H9.12c	mitochondrial cytochrome c-heme linkage protein Cyc2 (predicted)
SPAC17H9.14c	pdi2	protein disulfide isomerase
SPAC18G6.01c	SPAC18G6.01c	chalcone related protein family
SPAC18G6.02c	chp1	chromodomain protein Chp1
SPAC18G6.05c	SPAC18G6.05c	translation elongation regulator Gcn1 (predicted)
SPAC18G6.10	lem2	LEM domain protein Heh1/Lem2
SPAC18G6.12c	SPAC18G6.12c	ThiJ domain protein (predicted)
SPAC18G6.13	SPAC18G6.13	Schizosaccharomyces specific protein
SPAC19A8.04	erg5	C-22 sterol desaturase Erg5
SPAC1A6.04c	plb1	phospholipase B homolog Plb1

SPAC1B3.01c	SPAC1B3.01c	uracil phosphoribosyltransferase (predicted)
SPAC1B3.05	not3	CCR4-Not complex subunit Not3/5 (predicted)
SPAC1D4.03c	aut12	autophagy associated protein Aut12 (predicted)
SPAC20G4.07c	sts1	C-24(28) sterol reductase Sts1
SPAC20H4.03c	tfs1	transcription elongation factor TFIIS
SPAC20H4.04	fml2	ATP-dependent 3' to 5' DNA helicase (predicted)
SPAC20H4.05c	SPAC20H4.05c	5'-methylthioribulose-1-phosphate dehydratase, adducin (predicted)
SPAC20H4.06c	SPAC20H4.06c	RNA-binding protein
SPAC20H4.09	SPAC20H4.09	ATP-dependent RNA helicase, spliceosomal (predicted)
SPAC20H4.10	ufd2	ubiquitin-protein ligase E4 (predicted)
SPAC20H4.11c	rho5	Rho family GTPase Rho5
SPAC22H10.04	ppa3	protein phosphatase type 2A Ppa1
SPAC23C11.01	SPAC23C11.01	ER membrane protein, ICE2 family
SPAC23C11.06c	SPAC23C11.06c	hydrolase (inferred_from_context)
SPAC23C11.07	SPAC23C11.07	Schizosaccharomyces pombe specific protein
SPAC23C11.14	zhf1	zinc ion transporter Zhf1
SPAC23E2.01	fep1	iron-sensing transcription factor Fep1
SPAC23H3.11c	SPAC23H3.11c	glucosidase (predicted)
SPAC25A8.02	atg14	autophagy protein Atg14
SPAC32A11.02c	SPAC32A11.02c	conserved fungal protein
SPAC32A11.03c	phx1	stationary phase-specific homeobox transcription factor Phx1
SPAC3C7.01c	sac12	inositol polyphosphate phosphatase (predicted)
SPAC3C7.02c	pil2	meiotic eisosome BAR domain protein Pil2
SPAC3C7.04	SPAC3C7.04	transcription factor (predicted)
SPAC3C7.05c	mug191	alpha-1,6- mannanase (predicted)
SPAC3C7.06c	pit1	serine/threonine protein kinase, meiotic Pit1
SPAC3C7.07c	SPAC3C7.07c	arginine-tRNA protein transferase (predicted)
SPAC3C7.09	set8	lysine methyltransferase Set8 (predicted)
SPAC3C7.10	pex13	peroxin 13 (predicted)
SPAC3C7.12	tip1	CLIP170 family protein Tip1
SPAC3C7.13c	SPAC3C7.13c	glucose-6-phosphate 1-dehydrogenase (predicted)
SPAC3H1.06c	SPAC3H1.06c	membrane transporter (predicted)
SPAC3H1.10	pcs2	phytochelatin synthetase
SPAC3H1.12c	snt2	Lid2 complex subunit Snt2
SPAC3H1.13	ppk13	serine/threonine protein kinase Ppk13 (predicted)
SPAC3H1.14	SPAC3H1.14	cytoplasmic vesicle protein, Vid24 family (predicted)
SPAC4G9.02	rnh201	ribonuclease H2 complex subunit Rnh201 (predicted)
SPAC4G9.06c	chz1	histone chaperone Chz1 (predicted)
SPAC4G9.09c	arg11	N-acetyl-gamma-glutamyl-phosphate reductase/acetylglutamate kinase
SPAC4G9.13c	vps26	retromer complex subunit Vps26
SPAC4G9.14	SPAC4G9.14	mitochondrial Mpv17/PMP22 family protein 2 (predicted)
SPAC4G9.20c	SPAC4G9.20c	mitochondrial carrier with solute carrier repeats, organic acid transmembrane transporter (predicted)

SPAC56F8.02	SPAC56F8.02	AMP binding enzyme (predicted)
SPAC589.05c	SPAC589.05c	queuosine salvage protein
SPAC607.06c	SPAC607.06c	metallopeptidase (predicted)
SPAC607.07c	SPAC607.07c	Schizosaccharomyces specific protein
SPAC607.10	spo3	sporulation protein Spo3
SPAC631.01c	acp2	F-actin capping protein beta subunit Acp2
SPAC631.02	bdf2	BET family double bromodomain protein Bdf2
SPAC664.01c	swi6	HP1 family chromodomain protein Swi6
SPAC6C3.04	cit1	citrate synthase Cit1
SPAC6C3.05	SPAC6C3.05	Schizosaccharomyces specific protein
SPAC6C3.07	mug68	Schizosaccharomyces specific protein Mug68
SPAC6F12.06	SPAC6F12.06	Rho GDP dissociation inhibitor Rdi1 (predicted)
SPAC6F12.09	rdp1	RNA-directed RNA polymerase Rdp1
SPAC6G10.06	SPAC6G10.06	FAD-dependent amino acid oxidase involved in late endosome to Golgi transport (predicted)
SPAC732.02c	SPAC732.02c	fructose-2,6-bisphosphate 2-phosphatase activity (predicted)
SPAC767.01c	vps1	dynamin family protein Vps1
SPAC922.05c	SPAC922.05c	membrane transporter (predicted)
SPAC9G1.02	wis4	MAP kinase kinase kinase Wis4
SPAC9G1.04	oxa101	mitochondrial inner membrane translocase Oxa101
SPAC9G1.05	aip1	actin cortical patch component Aip1
SPAC9G1.06c	cyk3	cytokinesis protein Cyk3 (predicted)
SPAC9G1.07	SPAC9G1.07	Schizosaccharomyces specific protein
SPAC9G1.08c	SPAC9G1.08c	phospholipase (predicted)
SPAC9G1.10c	SPAC9G1.10c	inositol polyphosphate phosphatase (predicted)
SPAC9G1.11c	spn4	septin Spn4
SPAC9G1.12	cpd1	tRNA (m1A) methyltransferase complex catalytic subunit Cpd1
SPAP11E10.01	SPAP11E10.01	ornithine cyclodeaminase family (predicted)
SPAP14E8.04	oma1	metallopeptidase Oma1 (predicted)
SPAP27G11.02	SPAP27G11.02	TPR repeat protein, involved in mitochondrial protein turnover (predicted)
SPAPB1A10.05	SPAPB1A10.05	Schizosaccharomyces specific protein
SPAPB1A10.12c	alo1	D-arabinono-1,4-lactone oxidase (predicted)
SPAPB1A10.14	pof15	F-box protein (predicted)
SPBC1105.14	rsv2	transcription factor Rsv2
SPBC11G11.03	mrt4	mRNA turnover and ribosome assembly protein Mrt4 (predicted)
SPBC1604.12	SPBC1604.12	Schizosaccharomyces specific phosphoprotein
SPBC1652.02	SPBC1652.02	APC amino acid transporter (predicted)
SPBC1709.11c	png2	ING family homolog Png2
SPBC26H8.03	cho2	phosphatidylethanolamine N-methyltransferase Cho2
SPBC26H8.05c	pph3	serine/threonine protein phosphatase, PP4 complex subunit Pph3 (predicted)
SPBC2D10.17	clr1	cryptic loci regulator Clr1
SPBC2G2.03c	sbh1	translocon beta subunit Sbh1 (predicted)
SPBC30D10.10c	tor1	phosphatidylinositol kinase Tor1

SPBC409.07c	wis1	MAP kinase kinase Wis1
SPBC428.03c	pho4	thiamine-repressible acid phosphatase Pho4
SPBC428.06c	rxt2	histone deacetylase complex subunit Rxt2
SPBC543.03c	pku80	Ku domain protein Pku80
SPBC56F2.03	SPBC56F2.03	actin-like protein Arp10 (predicted)
SPCC126.15c	sec65	signal recognition particle subunit Sec65 (predicted)
SPCC1902.01	gaf1	transcription factor Gaf1
SPCC24B10.22	pog1	mitochondrial DNA polymerase gamma

Table S1B: Positive Interactors of *btn1*

ID	Name	Description
SPAC1006.03c	red1	RNA elimination defective protein Red1
SPAC1071.04c	spc2	signal peptidase subunit Spc2 (predicted)
SPAC1071.08	rpp203	60S acidic ribosomal protein A2
SPAC1142.07c	vps32	ESCRT III complex subunit Vps32
SPAC1142.08	fhl1	forkhead transcription factor Fhl1
SPAC11E3.08c	nse6	Smc5-6 complex non-SMC subunit Nse6
SPAC13G6.02c	rps101	40S ribosomal protein S3a
SPAC13G6.09	SPAC13G6.09	SSU-rRNA maturation protein Tsr4 homolog 2 (predicted)
SPAC144.04c	spe1	ornithine decarboxylase Spe1 (predicted)
SPAC144.11	rps1102	40S ribosomal protein S11 (predicted)
SPAC1486.01	SPAC1486.01	manganese superoxide dismutase (predicted)
SPAC1527.02	sft2	Golgi transport protein Sft2 (predicted)
SPAC1782.05	ypa2	protein phosphatase type 2A regulator, PTPA family Ypa2
SPAC1782.11	met14	adenylyl-sulfate kinase (predicted)
SPAC17A5.07c	ulp2	SUMO deconjugating cysteine peptidase Ulp2 (predicted)
SPAC17C9.14	SPAC17C9.14	Pex19 protein (predicted)
SPAC17G8.13c	mst2	histone acetyltransferase Mst2
SPAC1851.02	slc1	1-acylglycerol-3-phosphate O-acyltransferase Slc1 (predicted)
SPAC1851.03	ckb1	CK2 family regulatory subunit Ckb1
SPAC19B12.11c	SPAC19B12.11c	zinc finger protein, human ZNF593 ortholog
SPAC1B2.04	cox6	cytochrome c oxidase subunit VI (predicted)
SPAC1B3.16c	vht1	vitamin H transporter Vht1
SPAC1F12.07	SPAC1F12.07	phosphoserine aminotransferase (predicted)
SPAC20G4.04c	hus1	checkpoint clamp complex protein Hus1
SPAC222.08c	SPAC222.08c	glutamine aminotransferase subunit (predicted)
SPAC227.01c	erd1	Erd1 homolog (predicted)
SPAC227.05	SPAC227.05	prefoldin subunit 4 (predicted)
SPAC22A12.04c	rps2201	40S ribosomal protein S15a (predicted)
SPAC22F8.09	rrp16	rRNA processing protein Rrp16 (predicted)
SPAC22H10.11c	SPAC22H10.11c	TOR signaling pathway transcriptional corepressor Crf1 (predicted)

SPAC23C11.04c	pnk1	DNA kinase/phosphatase Pnk1
SPAC23C11.13c	hpt1	xanthine phosphoribosyltransferase (predicted)
SPAC23G3.05c	SPAC23G3.05c	regulator of G-protein signaling (RGS) domain (predicted)
SPAC23H3.13c	gpa2	heterotrimeric G protein alpha-2 subunit Gpa2
SPAC24B11.13	hem3	hydroxymethylbilane synthase Hem3 (predicted)
SPAC24H6.03	cul3	cullin 3
SPAC25A8.01c	fft3	SMARCAD1 family ATP-dependent DNA helicase Fft3
SPAC25G10.06	rps2801	40S ribosomal protein S28 (predicted)
SPAC25H1.07	emc1	ER membrane protein complex subunit Emc1 (predicted)
SPAC26A3.06	bud23	rRNA (guanine) methyltransferase Bud23 (predicted)
SPAC26H5.05	mga2	IPT/TIG ankyrin repeat containing transcription regulator of fatty acid biosynthesis (predicted)
SPAC30.02c	SPAC30.02c	elongator complex associated protein Kti2 (predicted)
SPAC30.03c	tsn1	translin
SPAC30C2.07	SPAC30C2.07	Schizosaccharomyces specific protein
SPAC31G5.11	pac2	cAMP-independent regulatory protein Pac2
SPAC31G5.17c	rps1001	40S ribosomal protein S10 (predicted)
SPAC323.05c	SPAC323.05c	protein methyltransferase Mtq2 (predicted)
SPAC328.10c	rps502	40S ribosomal protein S5 (predicted)
SPAC343.16	lys2	homoaconitate hydratase Lys2
SPAC343.19	lsb6	1-phosphatidylinositol 4-kinase Lsb6 (predicted)
SPAC3A12.10	rpl2001	60S ribosomal protein L20a (predicted)
SPAC3A12.13c	SPAC3A12.13c	translation initiation factor eIF3j (p35)
SPAC3G9.08	png1	ING family homolog Png1
SPAC3H8.05c	mms1	E3 ubiquitin ligase complex subunit Mms1 (predicted)
SPAC3H8.07c	pac10	prefoldin subunit 3 Pac10 (predicted)
SPAC4F10.19c	SPAC4F10.19c	zf-HIT protein Hit1 (predicted)
SPAC4G9.15	SPAC4G9.15	ketoreductase (predicted)
SPAC521.05	rps802	40S ribosomal protein S8 (predicted)
SPAC56F8.09	rrp8	rRNA methyltransferase Rrp8 (predicted)
SPAC57A10.12c	ura3	dihydroorotate dehydrogenase Ura3
SPAC5H10.06c	adh4	alcohol dehydrogenase Adh4
SPAC630.14c	tup12	transcriptional corepressor Tup12
SPAC637.10c	rpn10	19S proteasome regulatory subunit Rpn10
SPAC664.02c	arp8	actin-like protein, Ino80 complex subunit Arp8
SPAC6B12.02c	mus7	DNA repair protein Mus7/Mms22
SPAC6G10.08	idp1	isocitrate dehydrogenase Idp1 (predicted)
SPAC824.04	SPAC824.04	WD repeat protein (predicted)
SPAC890.05	SPAC890.05	ribosome biogenesis protein, G-patch domain, PINX1 family (predicted)
SPAC8C9.07	SPAC8C9.07	rRNA processing protein Fyv7 (predicted)
SPAC8C9.19	SPAC8C9.19	conserved fungal protein
SPAC8F11.02c	dph3	diphthamide biosynthesis protein Dph3 (predicted)
SPAC9.12c	atp12	F1-ATPase chaperone Atp12 (predicted)

SPAC9.13c	cwf16	splicing factor Cwf16
SPAC926.03	rlc1	myosin II regulatory light chain Rlc1
SPAC959.06c	SPAC959.06c	conserved fungal protein
SPAP27G11.14c	SPAP27G11.14c	Schizosaccharomyces pombe specific protein
SPAP7G5.05	rpl1002	60S ribosomal protein L10
SPAP8A3.07c	SPAP8A3.07c	phospho-2-dehydro-3-deoxyheptonate aldolase (predicted)
SPAPB1E7.06c	eme1	Holliday junction resolvase subunit Eme1
SPBC106.10	pka1	cAMP-dependent protein kinase catalytic subunit Pka1
SPBC106.17c	cys2	homoserine O-acetyltransferase (predicted)
SPBC119.12	rud3	Golgi matrix protein Rud3 (predicted)
SPBC1198.08	SPBC1198.08	dipeptidase Dug1 (predicted)
SPBC1271.15c	SPBC1271.15c	mitochondrial translation initiation factor IF-2Mt (predicted)
SPBC12C2.12c	glo1	glyoxalase I
SPBC12D12.09	rev7	DNA polymerase zeta Rev7 (predicted)
SPBC13G1.10c	mug81	ATP-dependent RNA helicase Slh1 (predicted)
SPBC1539.10	nop16	ribosome biogenesis protein Nop16 (predicted)
SPBC15C4.01c	oca3	TPR repeat protein Oca3/ ER membrane protein complex Ecm2 (predicted)
SPBC16D10.11c	rps1801	40S ribosomal protein S18 (predicted)
SPBC16E9.12c	pab2	poly(A) binding protein Pab2
SPBC16G5.15c	fkh2	forkhead transcription factor Fkh2
SPBC16H5.08c	SPBC16H5.08c	ribosome biogenesis ATPase, Arb family ABCF2-like (predicted)
SPBC1703.12	ubp9	ubiquitin C-terminal hydrolase Ubp9
SPBC1711.03	aim27	ER membrane protein complex subunit Aim27 (predicted)
SPBC1718.03	ker1	DNA-directed RNA polymerase I complex subunit Ker1
SPBC1734.12c	alg12	dolichyl pyrophosphate Man7GlcNAc2 alpha-1,6-mannosyltransferase Alg12 (predicted)
SPBC17A3.06	SPBC17A3.06	phosphoprotein phosphatase (predicted)
SPBC17D1.06	dbp3	ATP-dependent RNA helicase Dbp3 (predicted)
SPBC18H10.07	SPBC18H10.07	WW domain-binding protein 4 (predicted)
SPBC18H10.13	rps1402	40S ribosomal protein S14 (predicted)
SPBC19F8.08	rps401	40S ribosomal protein S4 (predicted)
SPBC19G7.17	SPBC19G7.17	translocon subunit Sec61 homolog (predicted)
SPBC1D7.04	mlo3	RNA binding protein Mlo3
SPBC20F10.05	nrl1	NRDE-2 family protein (predicted)
SPBC211.06	gfh1	gamma tubulin complex subunit Gfh1
SPBC215.02	bob1	prefoldin subunit 5 (predicted)
SPBC21B10.10	rps402	40S ribosomal protein S4 (predicted)
SPBC21B10.13c	yox1	MBF complex corepressor Yox1
SPBC21C3.20c	git1	C2 domain protein Git1
SPBC27B12.10c	tom7	mitochondrial TOM complex subunit Tom7 (predicted)
SPBC27B12.11c	pho7	transcription factor Pho7
SPBC28E12.04	SPBC28E12.04	Schizosaccharomyces specific protein
SPBC29A3.02c	his7	phosphoribosyl-AMP cyclohydrolase/phosphoribosyl- ATP pyrophosphohydrolase His7

SPBC2F12.09c	atf21	transcription factor, Atf-CREB family Atf21
SPBC2G5.02c	ckb2	CK2 family regulatory subunit Ckb2 (predicted)
SPBC2G5.06c	hmt2	sulfide-quinone oxidoreductase
SPBC30D10.16	pha2	phrenate dehydratase
SPBC30D10.18c	rpl102	60S ribosomal protein L10a
SPBC32F12.05c	cwf12	complexed with Cdc5 protein Cwf12
SPBC32F12.11	tdh1	glyceraldehyde-3-phosphate dehydrogenase Tdh1
SPBC32H8.07	git5	heterotrimeric G protein beta subunit Git5
SPBC337.04	ppk27	serine/threonine protein kinase Ppk27 (predicted)
SPBC337.09	erg28	Erg28 protein (predicted)
SPBC36.07	elp1	elongator subunit Elp1 (predicted)
SPBC3B9.13c	rpp102	60S acidic ribosomal protein A3
SPBC3D6.04c	mad1	mitotic spindle checkpoint protein Mad1
SPBC3E7.09	SPBC3E7.09	Sad1-UNC-like protein involved protein folding in the ER (predicted)
SPBC530.05	prt1	transcription factor (predicted)
SPBC56F2.08c	SPBC56F2.08c	RNA-binding protein (predicted)
SPBC609.05	pob3	FACT complex subunit Pob3
SPBC651.09c	prf1	RNA polymerase II associated Paf1 complex (predicted)
SPBC660.07	ntp1	alpha,alpha-trehalase Ntp1
SPBC6B1.06c	ubp14	ubiquitin C-terminal hydrolase Ubp14
SPBC725.01	SPBC725.01	aspartate aminotransferase (predicted)
SPBC725.14	arg6	acetylglutamate synthase Arg6 (predicted)
SPBC887.17	SPBC887.17	transmembrane transporter (predicted)
SPBC8D2.12c	SPBC8D2.12c	mitochondrial translational activator of cytochrome C oxidase I (predicted)
SPBC8D2.17	gmh4	alpha-1,2-galactosyltransferase (predicted)
SPBC8D2.18c	SPBC8D2.18c	adenosylhomocysteinase (predicted)
SPBP35G2.08c	air1	zinc knuckle TRAMP complex subunit Air1
SPBP4H10.13	rps2302	40S ribosomal protein S23 (predicted)
SPBP8B7.10c	utp16	U3 snoRNP-associated protein Utp16 (predicted)
SPBPB7E8.02	SPBPB7E8.02	PSP1 family protein
SPCC1020.06c	tal1	transaldolase (predicted)
SPCC1020.07	SPCC1020.07	haloacid dehalogenase-like hydrolase
SPCC1020.10	oca2	serine/threonine protein kinase Oca2
SPCC1020.11c	ecm6	ER membrane protein complex subunit 6 (predicted)
SPCC1183.02	SPCC1183.02	glutathione S-transferase (predicted)
SPCC11E10.03	mug1	dynactin complex subunit, dynamitin (predicted)
SPCC11E10.05c	ynd1	nucleoside diphosphatase Ynd1
SPCC11E10.06c	elp4	elongator complex subunit Elp4 (predicted)
SPCC11E10.08	rik1	silencing protein Rik1
SPCC11E10.09c	SPCC11E10.09c	alpha-amylase homolog (predicted)
SPCC1223.01	SPCC1223.01	ubiquitin-protein ligase E3 (predicted)
SPCC1223.06	tea1	cell end marker Tea1

SPCC1223.12c	meu10	GPI anchored cell surface protein involved in ascospore wall assembly Meu10
SPCC1235.05c	fft2	SMARCAD1 family ATP-dependent DNA helicase Fft2 (predicted)
SPCC1235.13	ght6	hexose transporter Ght6
SPCC1259.03	rpa12	DNA-directed RNA polymerase complex I subunit Rpa12
SPCC1259.08	SPCC1259.08	conserved fungal protein, DUF2457 family
SPCC1259.09c	pdx1	pyruvate dehydrogenase protein x component, Pdx1 (predicted)
SPCC1259.10	pgp1	mitochondrial metallopeptidase, tRNA N6-threonyl-carbamoyl-adenosine (t6A), a modification protein Pgp1
SPCC1259.11c	gyp2	GTPase activating protein Gyp2 (predicted)
SPCC1259.14c	meu27	UPF0300 family protein 5
SPCC1281.04	SPCC1281.04	pyridoxal reductase (predicted)
SPCC1281.07c	SPCC1281.07c	glutathione S-transferase (predicted)
SPCC1281.08	wtf11	wtf element Wtf11
SPCC132.01c	mtr1	microtubule regulator Mtr1
SPCC132.02	hst2	Sir2 family histone deacetylase Hst2
SPCC132.04c	gdh2	NAD-dependent glutamate dehydrogenase Gdh2 (predicted)
SPCC1322.02	pxd1	structure-specific DNA nuclease regulator Pxd1
SPCC1322.03	trp1322	TRP-like ion channel (predicted)
SPCC1322.06	kap113	karyopherin Kap113
SPCC1322.07c	mug150	Schizosaccharomyces pombe specific protein Mug150
SPCC1322.08	srk1	MAPK-activated protein kinase Srk1
SPCC1322.14c	vtc4	vacuolar transporter chaperone (VTC) complex subunit (predicted)
SPCC1322.15	rpl3402	60S ribosomal protein L34
SPCC1322.16	phb2	prohibitin Phb2 (predicted)
SPCC1393.10	ctr4	copper transporter complex subunit Ctr4
SPCC1393.13	SPCC1393.13	DUF89 family protein
SPCC1450.02	bdf1	Swr1 complex bromodomain subunit Brf1
SPCC162.10	ppk33	serine/threonine protein kinase Ppk33 (predicted)
SPCC162.12	tco89	TORC1 subunit Tco89
SPCC1672.06c	asp1	inositol hexakisphosphate kinase/inositol pyrophosphate synthase (predicted)
SPCC1682.14	rpl1902	60S ribosomal protein L19
SPCC16A11.01	SPCC16A11.01	plasma membrane protein involved in inositol lipid-mediated signaling Sfk1 (predicted)
SPCC16A11.03c	SPCC16A11.03c	DUF2009 protein
SPCC16A11.04	snx12	sorting nexin Snx12 (predicted)
SPCC16A11.07	coq10	mitochondrial ubiquinone binding protein Coq10
SPCC16A11.08	atg20	sorting nexin Atg20 (predicted)
SPCC16A11.16c	rpn1302	19S proteasome regulatory subunit Rpn13b
SPCC16C4.04	SPCC16C4.04	Schizosaccharomyces specific protein
SPCC16C4.14c	sfc4	transcription factor TFIIIC complex subunit Sfc4
SPCC16C4.17	mug123	Schizosaccharomyces specific protein Mug123
SPCC1739.06c	SPCC1739.06c	uroporphyrin methyltransferase (predicted)
SPCC1753.02c	git3	G-protein coupled receptor Git3
SPCC1753.03c	rec7	meiotic recombination protein Rec7

SPCC1753.05	rsm1	RNA export factor Rsm1
SPCC1795.03	gms1	UDP-galactose transporter Gms1
SPCC1795.09	yps1	aspartic protease, yapsin Yps1
SPCC1795.10c	SPCC1795.10c	Sed5 Vesicle Protein Svp26 (predicted)
SPCC18.17c	SPCC18.17c	proteasome assembly chaperone (predicted)
SPCC1840.03	sal3	karyopherin Sal3
SPCC188.02	par1	protein phosphatase regulatory subunit Par1
SPCC188.08c	ubp5	ubiquitin C-terminal hydrolase Ubp5
SPCC188.09c	pfl4	cell surface glycoprotein, adhesion molecule (predicted)
SPCC188.12	spn6	septin Spn6 (predicted)
SPCC18B5.03	wee1	M phase inhibitor protein kinase Wee1
SPCC18B5.06	dom34	peloto ortholog (predicted)
SPCC18B5.11c	cds1	replication checkpoint kinase Cds1
SPCC1906.02c	cue3	CUE domain protein Cue3 (predicted)
SPCC191.09c	gst1	glutathione S-transferase Gst1
SPCC1919.05	SPCC1919.05	Ski complex TPR repeat subunit Ski3 (predicted)
SPCC23B6.01c	SPCC23B6.01c	oxysterol binding protein (predicted)
SPCC23B6.02c	SPCC23B6.02c	pre-ribosomal factor (predicted)
SPCC23B6.03c	tel1	ATM checkpoint kinase
SPCC23B6.05c	ssb3	DNA replication factor A subunit Ssb3
SPCC24B10.02c	SPCC24B10.02c	NAD/NADH kinase (predicted)
SPCC24B10.10c	yta4	mitochondrial outer membrane ATPase Msp1/Yta4 (predicted)
SPCC24B10.11c	mft1	THO complex subunit 7 Mft1 (predicted)
SPCC24B10.13	skb5	Shk1 kinase binding protein 5
SPCC24B10.14c	xlf1	xrcc4 like factor, cernunnon
SPCC24B10.15	SPCC24B10.15	RNA endoribonuclease involved in mRNP quality control (predicted)
SPCC24B10.16c	SPCC24B10.16c	proteasome assembly chaperone 4 (predicted)
SPCC24B10.18	SPCC24B10.18	human Leydig cell tumor 10 kDa protein homolog
SPCC24B10.19c	nts1	Schizosaccharomyces specific protein
SPCC24B10.20	SPCC24B10.20	short chain dehydrogenase (predicted)
SPCC285.09c	cgs2	cAMP-specific phosphodiesterase Cgs2
SPCC285.15c	rps2802	40S ribosomal protein S28, Rps2802
SPCC285.17	spp27	RNA polymerase I upstream activation factor complex subunit Spp27
SPCC2H8.05c	dbl1	Schizosaccharomyces specific protein, double strand break localizing Dbl1
SPCC306.07c	SPCC306.07c	U3 snoRNP-associated protein Cic1/Utp30 family (predicted)
SPCC306.11	SPCC306.11	Schizosaccharomyces specific protein
SPCC31H12.02c	mug73	plasma membrane transmembrane transport regulator (predicted)
SPCC31H12.06	mug111	major facilitator family transmembrane transporter Mug111 (predicted)
SPCC320.03	SPCC320.03	transcription factor (predicted)
SPCC320.06	SPCC320.06	conserved fungal protein
SPCC320.14	sry1	serine racemase Sry1
SPCC330.02	rhp7	Rad7 homolog Rhp7

SPCC330.06c	pmp20	thioredoxin peroxidase Pmp20
SPCC330.07c	SPCC330.07c	membrane transporter (predicted)
SPCC330.11	btb1	BTB/POZ domain protein Btb1
SPCC330.12c	sdh3	succinate dehydrogenase (ubiquinone) cytochrome b subunit (predicted)
SPCC330.14c	rpl2402	60S ribosomal protein L24 (predicted)
SPCC338.02	mug112	Schizosaccharomyces pombe specific protein Mug112
SPCC338.04	cid2	caffeine induced death protein Cid2
SPCC338.11c	rrg1	protein-lysine N-methyltransferase Rrg1 (predicted)
SPCC338.16	pof3	F-box protein Pof3
SPCC338.18	SPCC338.18	Schizosaccharomyces pombe specific protein
SPCC417.07c	mto1	MT organizer Mto1
SPCC4B3.02c	SPCC4B3.02c	Golgi transport protein Got1 (predicted)
SPCC4B3.03c	SPCC4B3.03c	mitochondrial morphology protein (predicted)
SPCC4B3.06c	SPCC4B3.06c	NADPH-dependent FMN reductase (predicted)
SPCC4B3.07	nro1	negative regulator of Ofd1, Nro1
SPCC4B3.08	lsg1	Lsk1 complex gamma subunit (predicted)
SPCC4B3.11c	fra3	mitochondrial transcriptional repressor protein BolA domain (predicted)
SPCC4B3.12	set9	histone lysine H3-K20 methyltransferase Set9
SPCC4B3.13	SPCC4B3.13	MatE family transporter (predicted)
SPCC4G3.04c	coq5	C-methyltransferase (predicted)
SPCC4G3.10c	rhp42	DNA repair protein Rhp42
SPCC550.01c	coa4	mitochondrial respiratory chain complex assembly protein (predicted)
SPCC550.03c	SPCC550.03c	Ski complex RNA helicase Ski2 (predicted)
SPCC550.08	SPCC550.08	N-acetyltransferase (predicted)
SPCC550.09	SPCC550.09	peroxin Pex32 (predicted)
SPCC550.10	atd3	aldehyde dehydrogenase Atd3 (predicted)
SPCC550.11	SPCC550.11	karyopherin (predicted)
SPCC550.15c	SPCC550.15c	ribosome biogenesis protein (predicted)
SPCC553.01c	dbl2	meiotic chromosome segregation protein Dbl2
SPCC553.03	pex1	AAA family ATPase Pex1 (predicted)
SPCC553.12c	SPCC553.12c	transmembrane transporter (predicted)
SPCC584.03c	SPCC584.03c	Ran GTP-binding protein (predicted)
SPCC584.13	SPCC584.13	amino acid permease (predicted)
SPCC584.15c	SPCC584.15c	arrestin/PY protein 2
SPCC584.16c	SPCC584.16c	Schizosaccharomyces specific protein
SPCC594.01	SPCC594.01	DUF1769 family protein
SPCC594.02c	SPCC594.02c	conserved fungal protein
SPCC594.06c	SPCC594.06c	vacuolar SNARE Vam7 (predicted)
SPCC594.07c	bqt3	bouquet formation protein Bqt3
SPCC61.03	SPCC61.03	NADHX dehydratase (predicted)
SPCC61.05	SPCC61.05	S. pombe specific multicopy membrane protein family 1
SPCC613.01	SPCC613.01	membrane transporter (predicted)

SPCC613.02	SPCC613.02	membrane transporter (predicted)
SPCC613.06	rpl902	60S ribosomal protein L9
SPCC613.12c	raf1	CLRC ubiquitin E3 ligase complex specificiy factor Raf1/Dos1
SPCC622.01c	SPCC622.01c	dubious
SPCC622.08c	hta1	histone H2A alpha
SPCC622.12c	gdh1	NADP-specific glutamate dehydrogenase Gdh1 (predicted)
SPCC622.14	SPCC622.14	GTPase activating protein (predicted)
SPCC622.15c	SPCC622.15c	Schizosaccharomyces specific protein
SPCC622.16c	epe1	Jmjc domain chromatin associated protein Epe1
SPCC622.17	apn1	
SPCC622.19	jmj4	Jmj4 protein (predicted)
SPCC63.03	SPCC63.03	DNAJ domain protein, DNAJC11 family
SPCC63.04	mok14	alpha-1,4-glucan synthase Mok14
SPCC63.06	SPCC63.06	human WDR89 family WD repeat protein
SPCC63.14	eis1	eisosome assembly protein eis1
SPCC645.07	rgf1	RhoGEF for Rho1, Rgf1
SPCC645.08c	snd1	RNA-binding protein, involved in chromatin silencing by small RNA, Snd1
SPCC645.11c	mug117	conserved fungal protein Mug117
SPCC645.12c	SPCC645.12c	Schizosaccharomyces specific protein
SPCC645.13	SPCC645.13	transcription elongation regulator/ ubiquitin-protein ligase (predicted)
SPCC663.03	pmd1	leptomycin efflux transporter Pmd1
SPCC736.04c	gma12	alpha-1,2-galactosyltransferase Gma12
SPCC736.07c	SPCC736.07c	unconventional prefoldin involved in translation initiation (predicted)
SPCC736.09c	SPCC736.09c	TRAX
SPCC736.13	SPCC736.13	short chain dehydrogenase (predicted)
SPCC737.06c	SPCC737.06c	glutamate-cysteine ligase regulatory subunit (predicted)
SPCC74.02c	ppn1	PNUTS homologue, mRNA cleavage and polyadenylation specificity factor complex associated protein
SPCC757.05c	SPCC757.05c	peptidase family M20 protein
SPCC757.07c	ctt1	catalase
SPCC757.09c	rnc1	RNA-binding protein that suppresses calcineurin deletion Rnc1
SPCC757.12	SPCC757.12	alpha-amylase homolog (predicted)
SPCC777.03c	SPCC777.03c	nifs homolog, possible cysteine desulfurase
SPCC777.13	vps35	retromer complex subunit Vps35
SPCC777.15	SPCC777.15	tRNA dihydrouridine synthase Dus4 (predicted)
SPCC794.09c	tef101	translation elongation factor EF-1 alpha Ef1a-a
SPCC794.11c	ent3	ENTH/VHS domain protein Ent3 (predicted)
SPCC825.04c	naa40	histone N-acetyltransferase Naa40 (predicted)
SPCC825.05c	pwi1	splicing coactivator SRRM1 (predicted)
SPCC895.07	alp14	TOG ortholog Alp14
SPCC895.09c	ucp12	ATP-dependent RNA helicase Ucp12 (predicted)
SPCC962.04	rps1201	40S ribosomal protein S12 (predicted)
SPCC970.05	rpl3601	60S ribosomal protein L36

SPCP25A2.02c	rhp26	SNF2 family helicase Rhp26
SPCP31B10.07	eft202	translation elongation factor 2 (EF-2) Eft2,B
SPCPB16A4.02c	SPCPB16A4.02c	conserved fungal protein
SPCPB16A4.03c	ade10	bifunctional IMP cyclohydrolase/phosphoribosylaminoimidazolecarboxamideformyltransferase
SPCPB16A4.04c	trm8	tRNA (guanine-N7-)-methyltransferase catalytic subunit Trm8 (predicted)
SPCPB16A4.05c	SPCPB16A4.05c	urease accessory protein UREG (predicted)
SPCPB16A4.06c	SPCPB16A4.06c	Schizosaccharomyces specific protein
